# A dominant‐interfering *camta3* mutation compromises primary transcriptional outputs mediated by both cell surface and intracellular immune receptors in *Arabidopsis thaliana*

**DOI:** 10.1111/nph.14943

**Published:** 2017-12-11

**Authors:** Florence Jacob, Barbara Kracher, Akira Mine, Carolin Seyfferth, Servane Blanvillain‐Baufumé, Jane E. Parker, Kenichi Tsuda, Paul Schulze‐Lefert, Takaki Maekawa

**Affiliations:** ^1^ Department of Plant–Microbe Interactions Max Planck Institute for Plant Breeding Research 50829 Cologne Germany; ^2^ Institute of Plant Sciences Paris‐Saclay Centre National de la Recherche Scientifique Institut National de la Recherche Agronomique Université Paris‐Sud Université d'Evry Université Paris‐Diderot Sorbonne Paris‐Cité Université Paris‐Saclay 91405 Orsay France

**Keywords:** *Arabidopsis thaliana*, calmodulin‐binding transcription activator (CAMTA), cross‐tolerance, effector‐triggered immunity (ETI), immediate early genes, nucleotide‐binding domain and LRR‐containing proteins (NLRs), pattern‐triggered immunity (PTI), transcriptional responses

## Abstract

- Pattern recognition receptors (PRRs) and nucleotide‐binding domain and leucine‐rich repeat (LRR)‐containing proteins (NLRs) initiate pattern‐triggered immunity (PTI) and effector‐triggered immunity (ETI), respectively, each associated with the activation of an overlapping set of defence genes. The regulatory mechanism behind this convergence of PTI‐ and ETI‐mediated defence gene induction remains elusive.
- We generated transgenic Arabidopsis plants that enable conditional NLR activation without pathogen infection to dissect NLR‐ and PRR‐mediated transcriptional signals. A comparative analysis of over 40 transcriptome datasets linked calmodulin‐binding transcription activators (CAMTAs) to the activation of overlapping defence genes in PTI and ETI. We used a dominant *camta3* mutant (*camta3‐D*) to assess CAMTA functions in the corresponding transcriptional regulation.
- Transcriptional regulation by NLRs, although highly similar to PTI responses, can be established independently of pathogen‐associated molecular pattern (PAMP) perception, defence phytohormones and host cell death. Conditional expression of the N‐terminal coiled‐coil domain of the barley MLA (Mildew resistance locus A) NLR is sufficient to trigger similar transcriptional reprogramming as full‐length NLRs. CAMTA‐binding motifs are overrepresented in the 5′ regulatory regions of the identified primary immune response genes, consistent with their altered expression and disease resistance responses in *camta3‐D* plants.
- We propose that CAMTA‐mediated transcriptional regulation defines an early convergence point in NLR‐ and PRR‐mediated signalling.

## Introduction

Plants, unlike higher vertebrates, lack an adaptive immune system and thus rely on innate immunity to suppress pathogen growth. To mount inducible and local immune responses, the recognition of non‐self or modified‐self molecular structures is essential and is accomplished by two classes of immune receptor (Maekawa *et al*., 2011b). Extracellular perception of non‐self molecules is often mediated by plasma membrane‐resident pattern recognition receptors (PRRs) which detect widely conserved microorganism‐derived epitopes, so‐called pathogen/microbe‐associated molecular patterns (P/MAMPs) (Boller & Felix, 2009). The activation of a PRR by a P/MAMP is sufficient to induce defence responses limiting pathogen growth, and this mechanism is designated pattern‐triggered immunity (PTI). Host‐adapted pathogens intercept PTI by delivering virulence factors, termed effectors, into host cells, a subset of which targets PTI components. To counter this, plants have evolved an intracellular surveillance system for non‐self molecules that can activate immune responses despite a partially disabled PTI. Key components of intracellular non‐self detection are a family of nucleotide‐binding domain and leucine‐rich repeat (LRR)‐containing proteins (NLRs). These NLRs typically detect the presence or action of strain‐specific pathogen effectors, also called avirulence (Avr) effectors (Jones *et al*., 2016). The activation of effector‐triggered immunity (ETI) by NLRs suppresses pathogen growth. As infection attempts by avirulent pathogens normally co‐activate ETI and PTI, it is difficult to disentangle the relative contributions of PRR‐ and NLR‐derived signals to immune outputs.

ETI often results in a hypersensitive response (HR), a rapid and localized host cell death at sites of attempted pathogen invasion, whereas host cells retain viability during PTI on treatment with many characterized P/MAMPs. During PTI, a stereotypic set of immune‐associated physiological responses is induced within minutes of treatment with different P/MAMPs – including Ca^2+^ influx, extracellular alkalinization, a transient reactive oxygen species burst, mitogen‐activated protein kinase activation and ethylene (ET) production, followed by transcriptional reprogramming within 30 min (Boller & Felix, 2009). Because of the difficulty in discriminating NLR‐ from co‐activated PRR‐initiated immune responses on inoculation with avirulent pathogens, it remains unclear whether NLR‐mediated signalling converges with PRR‐triggered defence responses. On the basis of transcriptional profiles, it has been proposed that mainly temporal and quantitative differences account for distinct ETI and PTI outputs and that different NLRs trigger similar responses (Tao *et al*., 2003; Navarro *et al*., 2004; Tsuda & Katagiri, 2010). A highly overlapping gene set is induced in ETI and PTI (Tao *et al*., 2003; Navarro *et al*., 2004; Tsuda & Katagiri, 2010). In addition, a sustained increase in cytosolic Ca^2+^ and prolonged accumulation of reactive oxygen species have been reported in ETI in response to an avirulent pathogen (Grant *et al*., 2000). Calcium‐dependent protein kinases (CDPKs) are involved in two tested ETI‐associated immune responses and play a positive regulatory role in the onset of host cell death (Gao *et al*., 2013). Other components for Ca^2+^ signalling, such as calmodulin (CaM)‐binding transcription activator (CAMTA), have been linked to salicylic acid (SA)‐mediated immunity on the basis of age‐ or low temperature‐related *camta3* knockout autoimmune phenotypes (growth retardation and leaf lesions) (Du *et al*., 2009). However, it has been shown recently that constitutive immune activation in *camta3* knockout mutants is mainly a result of the ectopic activation of two NLRs (Lolle *et al*., 2017). Thus, the previously proposed physiological roles of CAMTA family members deduced from *camta3* knockout plants need to be reconsidered.

Plant NLRs are subdivided into two classes, TNLs and CNLs, based on the presence of either a Toll‐interleukin 1 receptor (TIR) domain or a coiled‐coil (CC) domain at their N‐terminus, respectively (Maekawa *et al*., 2011b). NLR proteins are structurally conserved and also function as intracellular innate immune sensors for non‐self recognition in metazoans. For plant NLRs, the N‐terminal TIR and CC domains are thought to function as signal emitters and as facilitators of hetero‐ or homodimeric receptor complex formation, but, unlike in metazoans, there are no clear indications of oligomeric plant NLR complexes with defined stoichiometry (Hu *et al*., 2015; Zhang *et al*., 2015).

In this study, we first uncoupled NLR‐ and PRR‐mediated immune signals during ETI on inoculation with avirulent pathogens by conditional expression of the barley Mildew resistance locus A CC (MLA_CC_) domain in Arabidopsis or temperature‐induced activation of the Arabidopsis full‐length TNL RPS4 (RESISTANT TO *P. SYRINGAE* 4) (Maekawa *et al*., 2011a; Heidrich *et al*., 2013; Sohn *et al*., 2014). We then interrogated the role of the CAMTA protein family in defence gene reprogramming. We provide evidence that CAMTAs contribute to primary transcriptional responses in both PTI and ETI, and these are tightly associated with PRR‐ and NLR‐mediated disease resistance. Our work identifies the CAMTA‐regulated machinery as an early PRR and NLR post‐activation signalling event.

## Materials and Methods

### Comparative transcriptomics analysis

**Figure 1:**
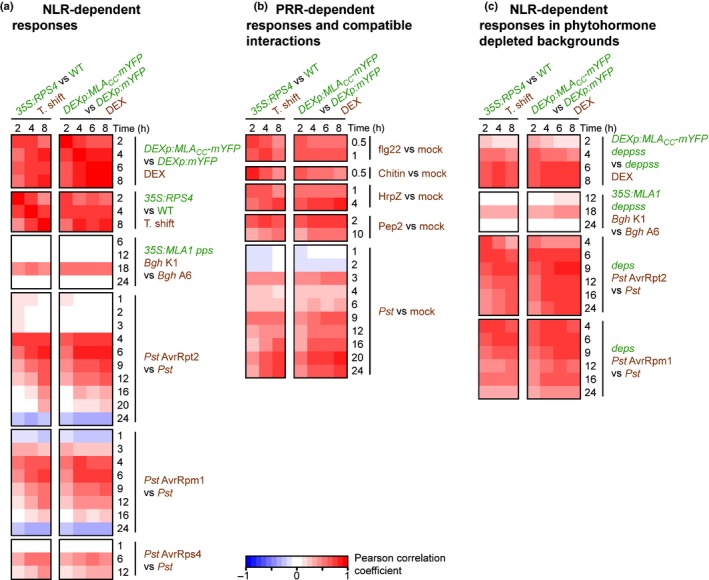
Transcriptome‐wide correlation analysis between effector‐triggered immunity (ETI), pattern‐triggered immunity (PTI) and non‐immune‐related responses in *Arabidopsis thaliana*. Time‐resolved nucleotide‐binding domain and leucine‐rich repeat (LRR)‐containing protein (NLR)‐dependent transcriptional responses mediated by conditional activation of MLA_CC_ (Mildew resistance locus A coiled‐coil) and RPS4 (RESISTANT TO *P. SYRINGAE* 4) in a pathogen‐free system were compared with responses to various other immune‐related stimuli. (a) Comparison with other NLR‐dependent responses. MLA1 and RPS4 encode a CNL and a TNL recognizing effectors of *Blumeria graminis* f. sp. *hordei* isolate K1 (*Bgh* K1) and *Pseudomonas syringae* pv. *tomato* DC3000 AvrRps4 (*Pst* AvrRps4), respectively. The AvrRpt2 and AvrRpm1 effectors are recognized by the CNLs RPS2 and RPM1, respectively, in wild‐type Arabidopsis. (b) Comparison with PTI‐related responses triggered by inoculation with a virulent pathogen (*Pst*) or treatment with a purified pathogen‐associated molecular pattern (PAMP) (flg22, chitin and HrpZ) or damage‐associated molecular pattern DAMP (Pep2). (c) Comparison with NLR‐dependent responses in phytohormone signalling‐compromised mutants. The *deps* and *deppss* mutants are deficient in phytohormone signalling pathways mediated by salicylic acid (SA), ethylene (ET) and jasmonic acid (JA). (a–c) Pearson correlations between different conditions were calculated on the basis of the relative expression (log_2_ fold change (log_2_ FC) (treatment/control)) values of all commonly expressed genes. The applied treatment is indicated in brown, and any genotypes different from Col‐0 are indicated in green. DEX, dexamethasone.

For comparative transcriptomics analysis of ETI‐ and PTI‐related responses in Arabidopsis, we combined published and unpublished data (Supporting Information Table S1). To minimize potentially confounding factors from organ‐ and developmental stage‐specific transcripts, we included mainly datasets obtained from mature rosette leaves (Fig. 1). Raw expression data from each experiment were normalized and re‐analysed with the same method, and the global similarity of the expression patterns was examined in a pairwise manner using Pearson's correlation coefficients (*r*) based on the log_2_ fold change (log_2_FC) values of all commonly expressed genes. Further methods related to RNA‐seq data acquisition (including pathogen inoculation) and transcriptomic analysis are described in Methods S1.

### Molecular cloning of MLA variants

All cloning reactions were performed using the Gateway technology according to the manufacturer's instructions. Dexamethasone‐inducible expression of the monomeric yellow fluorescent protein (mYFP) and MLA_CC_ variants with C‐terminally fused mYFP was achieved by transferring the corresponding cDNA fragments described in Maekawa *et al*. (2011a) into a Gateway technology‐compatible pTA7002 (Aoyama & Chua, 1997), in which the Gateway cassette (Thermo Fisher Scientific, Waltham, MA, USA) was inserted between the *Spe*I and *Xho*I restriction sites. Expression vectors for the full‐length MLA1 variants carrying either the autoactive mutation (D502V: GAT to GTT) or the P‐loop mutation (K207R: AAG to AGG) were generated using a QuikChange II site‐directed mutagenesis kit (Agilent Technologies, Santa Clara, CA, USA), and the sequence for the C‐terminal 3xTy1 epitope tag (5′‐AAGGGTGGGCGCGCCGAGGTGCACACCAACCAGGACCCCCTGGACGCCGAAGTCCATACAAATCAGGATCCTCTGGATGCCGAAGTGCACACCAATCAGGATCCCCTGGACGCTTAG‐3′) was introduced by PCR. The resulting cDNA fragments in pENTR vectors were transferred into the Gateway technology‐compatible pTA7002.

### Plant material and growth conditions

The *Arabidopsis thaliana* (L.) Heynh. ecotype Columbia (Col‐0) was used in this study. The mutant plants used here were *dde2‐2 ein2‐1 pad4‐1 pen2‐1 sag101‐2 sid2‐2* (*deppss*, Maekawa *et al*., 2012), *rpm1‐3 rps2‐101C* (*rpm1 rps2*, Mackey *et al*., 2003), *camta3‐1* (*camta3‐KO*, Galon *et al*., 2008) and *sr1‐4D* (*camta3‐D*, Nie *et al*., 2012). The *35S:MLA1‐HA dde2‐2 ein2‐1 pad4‐1 pen2‐1 sag101‐2 sid2‐2* (*35S:MLA1 deppss*) and *35S:RPS4‐HS* transgenic lines have been described previously (Maekawa *et al*., 2012; Heidrich *et al*., 2013). *Agrobacterium tumefaciens* strain GV3101 (pMP90RG, Koncz & Schell, 1986) was used to generate transgenic lines carrying the expression constructs. The *DEXp:MLA*_*CC*_ *‐mYFP deppss* line was obtained by transforming DDE2/*dde2‐2 ein2‐1 pad4‐1 pen2‐1 sag101‐2 sid2‐2* mutant plants and subsequently selecting T_2_ plants with the homozygous *dde2‐2* mutation. We confirmed that more than two independent transgenic lines in the T_1_ generation exhibited the same cell death‐inducing phenotype on dexamethasone application. For each construct, transgenic lines displaying a segregation of the selection marker (i.e. hygromycin resistance) consistent with a single transgene insertion in the T_2_ generation were selected (except for the MLA1 expressing lines). T_3_ generations homozygous for the transgene were used for the experiments. The *DEXp:MLA*_*CC*_ *‐mYFP camta3‐D* line was obtained by crossing the homozygous *DEXp:MLA*_*CC*_ *‐mYFP* line in the wild‐type with *camta3‐D* plants. *camta3‐D* plants were used as female parents. The first generation after crossing (F_1_) was used for the experiment.

Seedlings were initially grown on Murashige and Skoog (MS)‐agar plates for 2 wk in a growth cabinet (10 h : 14 h, light : dark cycle at 22°C) and subsequently transferred to 42‐mm Jiffy pots (Jiffy, Kristiansand, Norway) rehydrated in water with 0.1% fertilizer Wuxal TopN (Aglukon, Düsseldorf, Germany). Plants were grown for two more weeks under short‐day conditions in a growth chamber (Snijders Labs, Tilburg, the Netherlands; 10 h : 14 h, light : dark cycle at 22°C, 60% relative humidity). Four‐ to 5‐wk‐old plants were used for all analyses unless otherwise stated.

### Growth of Arabidopsis lines expressing dexamethasone‐inducible transgenes on dexamethasone‐containing agar plates

Surface‐sterilized seeds were sown onto round Petri dishes (9.2 cm in diameter) containing half‐strength MS‐agar medium with or without 10 μM dexamethasone (D1756‐1G, Sigma‐Aldrich, St Louis, MO, USA), and placed for 3 d at 4°C before transfer to a growth cabinet (10 h : 14 h, light : dark cycle at 22°C). Photographs were taken 16 d after germination with a CCD Color digital camera ProgRes C7 (Jenoptik, Jena, Germany) using ProgRes Capture Pro 2.10.0 software.

### Ion leakage assay following *Pseudomonas syringae* pv. *tomato* DC3000 (*Pst*) or dexamethasone infiltration

For each experiment, three sets of four leaf discs (5 mm in diameter) from at least five independent plants were sampled from infiltrated leaves with a biopsy punch, 20 min after infiltration, rinsed briefly in Milli‐Q water, dried on paper, transferred to three wells of a 24‐well plate each containing 1 ml Milli‐Q water with 0.001% Silwet L‐77 (Lehle Seeds, Round Rock, TX, USA), and incubated at 20°C for the time of the experiment. The conductivity was measured over time using a LAQUAtwin COND apparatus (Horiba, Kyoto, Japan). The experiment was repeated at least three times.

### Trypan blue staining

Staining with lactophenol–trypan blue has been described previously (Maekawa *et al*., 2012).

### Immunoblot assays

Leaf material from at least five independent plants was sampled at the indicated time point, frozen in liquid nitrogen and homogenized using an MM400 tissue lyser (Retsch, Haan, Germany) and steel beads. The proteins were extracted in 2 × sodium dodecylsulfate (SDS) sample buffer, separated by sodium dodecylsulfate‐polyacrylamide gel electrophoresis (SDS‐PAGE) and electro‐blotted onto Immobilon‐P poly(vinylidene difluoride) (PVDF) transfer membranes (Merck, Darmstadt, Germany). Equal protein transfer was monitored by staining membranes with Ponceau S. The membranes were subsequently blocked for 3 h in tris buffered saline with 0.1% tween 20 (TBS‐T) with 5% w/v nonfat dry milk before overnight incubation at 4°C with the corresponding primary antibody in TBS‐T with 5% w/v nonfat dry milk. The appropriate horseradish peroxidase (HRP)‐conjugated secondary antibody was applied for 2 h in TBS‐T with 5% w/v nonfat dry milk. Membrane detection was performed using Pico or Femto chemiluminescence reagent (Thermo Fisher Scientific) and the ChemiDoc MP imaging system (BioRad, Hercules, CA, USA). For protein quantification, the membranes were stained with Coomassie brilliant blue. Primary antibodies were monoclonal antibodies from mouse: α‐TY1 (SAB4800032, 1 : 1000, Sigma‐Aldrich), α‐FLAG (F1804, 1 : 5000, Sigma‐Aldrich) or α‐GFP (JL‐8, 1 : 5000, Takara, Shiga, Japan). As secondary antibody, a goat α‐mouse IgG‐HRP antibody was used (1 : 10000, Santa Cruz Biotechnology, Dallas, TX, USA).

### Quantification of CAMTA3‐FLAG steady‐state level from immunoblot assays

Images were taken with a ChemiDoc MP imaging system (BioRad) and analysed with Image Lab software (BioRad). All time course samples in an experiment were examined on the same blot. The band intensity of CAMTA3‐FLAG was detected using immunoblotting, whereas the amount of total protein in each sample was obtained by Coomassie brilliant blue staining following immunodetection. The relative band intensity of CAMTA3‐FLAG at a given time point was determined as the ratio of the band intensity to the highest signal value within the same blot. The relative band intensity at each time point was further adjusted by the amount of total proteins in the lane. The resulting protein levels from six independent experiments were standardized.

## Results

### Early convergence of NLR‐ and PRR‐mediated signalling at the transcriptome level

For comparative transcriptome analysis of ETI‐ and PTI‐related responses in Arabidopsis, we combined published and unpublished data (Table S1). To minimize potentially confounding factors from organ‐ and developmental stage‐specific transcripts, we included mainly datasets obtained from mature rosette leaves (Fig. 1, see the Materials and Methods section).

To uncouple co‐activated PTI and ETI responses on inoculation with avirulent pathogens, we utilized transgenic Arabidopsis lines enabling conditional NLR activation under pathogen‐free conditions (P‐FCs). Time‐resolved expression profiles obtained from these P‐FCs were then compared with time‐resolved transcriptional changes following leaf inoculation with avirulent pathogens or leaf treatment with PAMPs (Fig. 1), the latter probably activating PRR signalling only.

We chose to synchronously activate NLR responses across all leaf tissues mediated by the TNL‐type RPS4 or CNL‐type MLA receptor under P‐FCs. Conditional RPS4 activation is achieved by shifting plants that constitutively express the receptor from 28°C to 19°C, thereby triggering transcriptional changes in the absence of a pathogen (Heidrich *et al*., 2013). Similar to Col‐0 wild‐type plants, in which RPS4 mediates immunity with low host cell death to pathogenic *Pst* expressing AvrRps4, cell death was undetectable until 4 d after temperature shift‐mediated RPS4 activation, excluding the possibility that additional cell death‐associated cues complicate the interpretation of receptor‐mediated transcriptional reprogramming.

To compare RPS4‐mediated transcriptional changes with the MLA‐triggered response, we generated transgenic Arabidopsis lines that conditionally express either the MLA CC domain (MLA_CC_) fused to a C‐terminal mYFP, or mYFP alone, under a dexamethasone‐inducible promoter (Fig. S1a–e). Expression of MLA_CC_ in stable Arabidopsis transgenic lines results in severe growth defects (Fig. S1a,b). The MLA_CC_‐mYFP fusion protein was detectable as early as 2 h post‐induction, whereas cell death, monitored by ion leakage assays and trypan blue staining, was detectable from 4 h post‐induction onwards (Fig. S1c,e). Strikingly, the comparison of expression profiles between MLA_CC_‐ and RPS4‐mediated responses revealed a pronounced positive correlation under the tested P‐FCs (Fig 1a, 0.56 < *r *<* *0.83), implying that the two signalling pathways converge at the transcriptional level independent of cell death.

Next, we compared both RPS4 and MLA_CC_ transcriptional outputs in P‐FCs with gene expression patterns in response to leaf inoculations with four different avirulent pathogens (Fig. 1a). The strongest positive correlation between RPS4 profiles in P‐FCs and the expression profile mediated by full‐length MLA1 occurs specifically at 18 h post‐inoculation of the *Blumeria graminis* f. sp. *hordei* (*Bgh*) pathogen (0.44 < *r *<* *0.55), contrasting with the other tested time points (6, 12 and 24 h post‐inoculation: −0.09 < *r *<* *0.09). This is consistent with a previous RNA‐seq study, in which major MLA1‐dependent transcriptional changes were detected exclusively at 18 h post‐inoculation in Arabidopsis in response to an avirulent powdery mildew strain (Maekawa *et al*., 2012). The response mediated by conditional RPS4 activation was also similar to the transcriptional outputs triggered by *Pst* expressing AvrRpt2 (*Pst* AvrRpt2) (at 4–6 h post‐infiltration (hpi): 0.50 < *r *<* *0.86), *Pst* AvrRpm1 (at 4–6 hpi: 0.42 < *r *<* *0.74) and, to a lesser extent, *Pst* AvrRps4 (at 6–12 hpi: 0.16 < *r *<* *0.53) (Fig. 1a). An overlap between the RPS4 transcriptional output induced by either *Pst* AvrRps4 or temperature shift has been documented previously (Heidrich *et al*., 2013; Sohn *et al*., 2014). Interestingly, under P‐FCs, the RPS4 transcriptional response at 2 h post‐induction is similar to the early ETI profiles mediated by the CNL‐type RPS2 and RPM1 receptors at 4 hpi (*r *=* *0.72 and 0.56, respectively), but barely correlated with the same ETI profiles at later time points, such as 16 hpi (*r *=* *0.050 and 0.047, respectively). This suggests that, at early time points, conditional RPS4 activation in P‐FCs resembles an authentic early ETI‐related transcriptional response. A clear positive correlation was also detected between the MLA_CC_‐mediated response (4 h post‐induction) and the pathogen‐triggered transcriptional responses mediated by RPS2 (4 hpi: *r *=* *0.77), RPM1 (4 hpi: *r *=* *0.72) and MLA1 (18 h post‐inoculation: *r *=* *0.57) (Fig. 1a). This observation is consistent with the proposed role of the CC moiety as a signal emitter in the context of activated full‐length barley MLA and wheat Sr33, an orthologue of barley MLA (Maekawa *et al*., 2011a; Casey *et al*., 2016).

We next compared RPS4 and MLA_CC_ transcriptional outputs under P‐FCs with a set of early transcriptional responses to diverse PAMPs (flg22, chitin and HrpZ) and a DAMP (damage‐associated molecular pattern; Pep2), which are recognized extracellularly by corresponding membrane‐resident PRRs (Couto & Zipfel, 2016; Fig. 1b; Table S1). There was strong similarity between conditional RPS4 and MLA_CC_ transcriptional outputs and these PRR‐mediated responses (Fig. 1b). The highest correlation value observed in these comparisons with the various PRR‐dependent expression changes was 0.81 (for HrpZ treatment at 4 h and chitin octamer treatment at 0.5 h compared with the MLA_CC_‐ and RPS4‐mediated responses at 8 and 2 h post‐induction, respectively). As conditional expression of RPS4 or MLA_CC_ mimics ETI‐associated transcriptional outputs, these data, together with previous studies (Eulgem *et al*., 2004; Navarro *et al*., 2004), imply that NLR‐ and PRR‐triggered immunity induce a qualitatively similar response at the transcriptome level, and that the NLR‐mediated response can occur independently of P/DAMP perception.

We also compared RPS4‐ and MLA_CC_‐conditioned transcriptional outputs under P‐FCs with a time course experiment of a compatible interaction with virulent *Pst* (Fig. 1b). The highest positive correlation was detected at 20 hpi (*r *=* *0.81), which is much later than for the tested host interactions with avirulent *Pst* with maxima at 4–6 hpi (Fig. 1a). Notably, the strength of the correlation decreased at 4–6 hpi and increased again after 9 hpi (Fig. 1b). This contrasts with the transcriptional patterns seen across the tested host interactions with avirulent pathogens, showing a single maximum for the correlation coefficient (Fig. 1a). This difference may partly reflect the fact that pathogen growth is rapidly attenuated in ETI, whereas virulent pathogens suppress host defence and proliferate during compatible interactions.

Previous studies have shown a differential requirement for defence phytohormones in CNL‐mediated ETI in Arabidopsis (Tsuda *et al*., 2009; Maekawa *et al*., 2012; Cui *et al*., 2017). In quadruple mutant plants that simultaneously lack ET, jasmonic acid (JA) and SA signalling, designated *deps* (*dde2 ein2 pad4 sid2*), RPS2‐mediated pathogen growth restriction was impaired by 80%, whereas RPM1‐ or MLA1‐mediated immunity was largely retained (Tsuda *et al*., 2009; Maekawa *et al*., 2012). These observations prompted us to compare RPS4‐ and MLA_CC_‐conditioned transcriptomic data under P‐FCs with expression profiles during MLA1‐, RPS2‐ and RPM1‐mediated ETI in the *deps* quadruple mutant background (Tsuda *et al*., 2009). As the Arabidopsis *pen2 pad4 sag101* triple mutant is needed to see a strong susceptible phenotype to non‐adapted barley powdery mildew (Lipka *et al*., 2005), we generated sextuple mutant plants, designated *deppss* (*dde2 ein2 pad4 pen2 sid2 sag101*), which are additionally impaired in each of the three aforementioned defence phytohormone signalling pathways, to examine MLA1‐mediated immunity and the MLA_CC_‐elicited response in this background (Fig. 1c).

The correlation patterns in the mutant backgrounds were similar overall to those observed when comparing the same responses in wild‐type backgrounds (Fig. 1c). However, the correlation values on conditional MLA_CC_ expression at 2 h post‐induction and the response on *Pst* AvrRpt2 challenge at 4 hpi were lower in the defence phytohormone‐depleted backgrounds than in the wild‐type (at 2 h post‐induction: 0.56 < *r *<* *0.76 and 0.16 < *r *<* *0.26 for MLA_CC_ in wild‐type and *deppss*, respectively; at 4 h post‐induction: 0.71 < *r *<* *0.78 and 0.36 < *r *<* *0.71 for *Pst* AvrRpt2 in wild‐type and *deps*, respectively; as compared with RPS4 outputs in P‐FCs, Fig. 1c). We cannot exclude the possibility that the lower steady‐state level of MLA_CC_ protein in the *deppss* background contributes to the reduced correlation of transcriptional outputs (Fig. S1d). Furthermore, unlike the expression profiles in wild‐type plants, the profiles of both RPS4‐ and MLA_CC_‐conditioned responses under P‐FCs were still positively correlated with the ETI profiles mediated by RPS2 and RPM1 at 16 hpi or later in the *deps* background (Fig. 1c). Despite the slight qualitative and temporal differences, these results indicate that the signal transduction needed for convergent transcriptional responses downstream of various PRRs and NLRs is largely retained, even on simultaneous impairment of SA, JA and ET signalling during CNL‐triggered ETI.

### Transcriptional upregulation during early ETI, PTI and abiotic stress responses

Comparative analyses of time‐resolved transcriptome profiles after conditional activation of RPS4 and MLA_CC_ showed that the corresponding gene expression patterns at 2 h post‐induction resemble early ETI and PTI transcriptional outputs (Fig. 1). At this time point, 1076 and 562 genes are significantly upregulated, whereas 247 and 11 genes are downregulated on temperature‐conditioned RPS4 activation and MLA_CC_ expression, respectively (|log_2_FC| > 1 and false discovery rate (FDR) < 0.01, Fig. S2). As transcriptional upregulation is generally observed early in ETI and PTI (|log_2_FC| > 1 and FDR < 0.01, Fig. S2), the induction of a defined set of genes appears to be a principal feature of these early immune responses.

**Figure 2:**
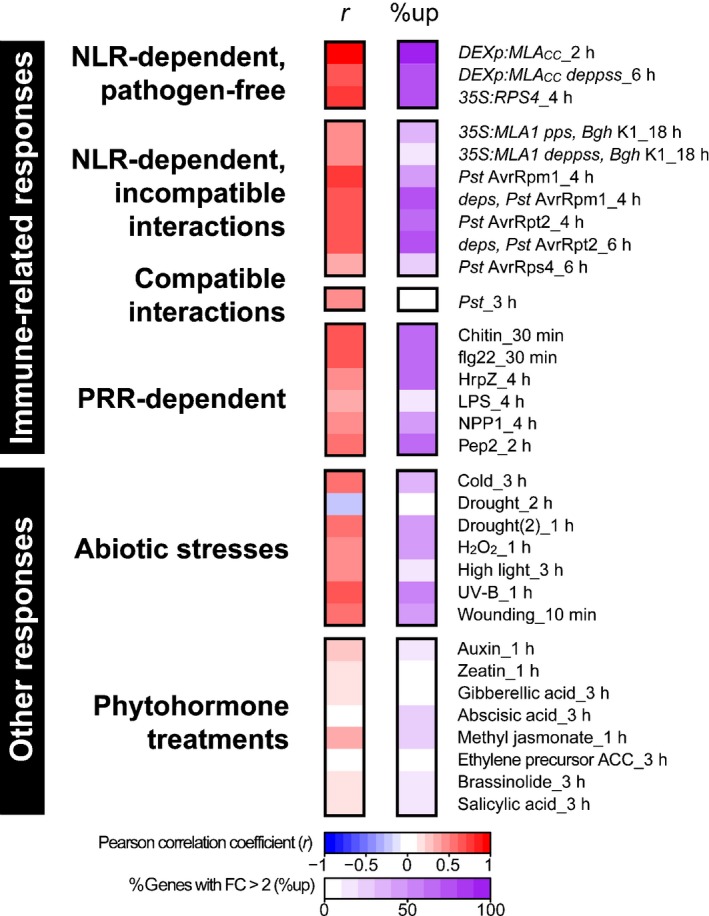
Expression profiles of *Arabidopsis* genes rapidly induced on conditional MLA_CC_ (Mildew resistance locus A coiled‐coil) expression during early responses to various stimuli. The analysis is based on 478 of the 562 genes induced by MLA_CC_ at 2 h post‐induction (excluding genes unavailable in ATH1 22K microarray‐based experiments). The left panel represents the Pearson correlation (*r*) of log_2_ fold change (log_2_ FC) (treatment/control) values between conditional MLA_CC_ expression at 2 h post‐induction and the other treatments. The right panel indicates, for each treatment, the proportion of these 478 genes with more than two‐fold induction (%up). The data shown here are a representative subset of the dataset shown in Supporting Information Fig. S3.

To obtain a broader view, we examined patterns of transcriptional upregulation on various stimuli, including abiotic stresses (Figs 2, S3; Tables S1, S2). As the computed correlation coefficients reflect qualitative similarities, but do not account well for quantitative differences, we additionally calculated the proportion of induced genes (%up : %genes with log_2_FC > 1) in each dataset. For simplicity, we chose the dataset obtained on expression of MLA_CC_ at 2 h post‐induction for subsequent comparisons. We restricted this analysis to 478 of the 562 upregulated genes, because data for the other 84 genes are unavailable in ATH1 22K microarray‐based experiments. To highlight the proportion of induced genes in response to the different stimuli, we selected the time point that exhibited the highest proportion of induced genes for each time‐resolved response profile (Fig. 2).

On inoculation with avirulent pathogens, many, but not all, of the 478 MLA_CC_‐induced genes were induced, irrespective of the presence/absence of functional ET, JA and SA signalling pathways (e.g. 50%up in response to *Pst* AvrRpm1 in wild‐type against 71%up in response to *Pst* AvrRpm1 in *deps*, Fig. 2). In contrast with the incompatible interactions, and despite a positive correlation coefficient observed for the expression changes, very few of the 478 upregulated genes were induced in the compatible interaction on challenge with virulent *Pst* throughout the examined time frame (at most 3%up at 3 hpi, Figs 2, S3). Although a different set of genes was examined, a similar quantitative difference was also observed between incompatible and compatible interactions in an earlier study (Tao *et al*., 2003). Considering that application of PAMPs also induced a major part of this gene set (e.g. 62%up in response to chitin at 0.5 h post‐treatment and 65%up in response to flg22 at 0.5 h post‐treatment, Fig. 2), the virulent pathogen, although harbouring PAMPs, appears to suppress the induction of these genes in the compatible interaction, presumably by delivering effectors into host cells.

Interestingly, some of the 478 MLA_CC_‐induced genes were also upregulated in response to a range of abiotic stresses and, consistent with this, correlation coefficients for the corresponding expression changes indicate a positive correlation (0.44 < *r *<* *0.67, Figs 2, S3). Importantly, despite the well‐established roles of the phytohormones abscisic acid (ABA), JA, ET and SA in abiotic and biotic stress responses (Grosskinsky *et al*., 2016), the application of these phytohormones alone did not strongly induce the expression of these genes (Figs 2, S3). This finding suggests that these phytohormones play minor roles in the early transcriptional changes accompanying abiotic and biotic stress responses.

### Induction of common immediate‐early (IE) genes in ETI and PTI

**Figure 3:**
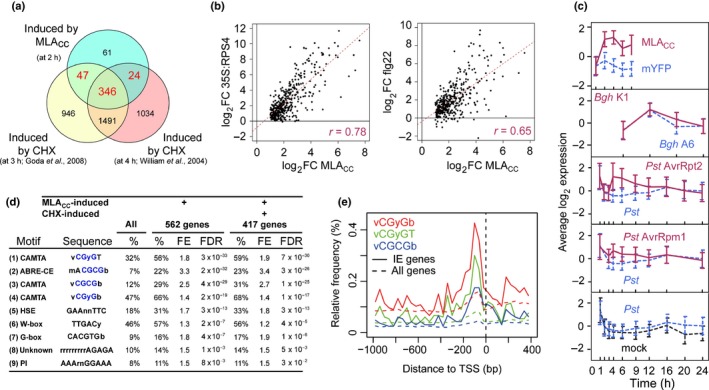
Induction of common immediate‐early (IE) genes in effector‐triggered immunity (ETI) and pattern‐triggered immunity (PTI) in *Arabidopsis thaliana*. (a) Overlap between the gene sets induced by MLA_CC_ (Mildew resistance locus A coiled‐coil) at 2 h post‐induction (false discovery rate (FDR) < 0.01 and fold change (FC) > 2) and by two independent cycloheximide (CHX) treatments for 3 and 4 h in seedlings (FDR < 0.05 and FC > 1). The intersection between the gene sets induced by MLA_CC_ at 2 h post‐induction and at least one of the CHX treatments defines a set of 417 IE genes that are activated by both stimuli. The analysis was restricted to 478 of the 562 MLA_CC_‐induced genes, as the other 84 genes are unavailable in ATH1 22K microarray‐based experiments. (b, c) Expression changes of the 417 IE genes in other ETI and PTI responses. (b) Scatter plots showing the expression changes of the 417 IE genes on MLA_CC_ expression at 2 h post‐induction in comparison with the changes induced on conditional activation of the RPS4‐mediated response at 2 h post‐induction (left plot) and on flg22 treatment for 0.5 h (right plot). The corresponding Pearson correlations are indicated in magenta. (c) Time‐resolved expression profiles of the 417 IE genes during several CNL‐dependent ETI responses (plain magenta lines), compatible interactions (dashed blue lines) and respective mock treatments (dashed black lines). The data represent mean ± SD. *Bgh*,* Blumeria graminis* f. sp. *hordei*; mYFP, monomeric yellow fluorescent protein; *Pst*,* Pseudomonas syringae* pv. *tomato*. (d) Motifs over‐represented in the 5′ *cis*‐regulatory regions of all 562 genes induced by MLA_CC_ at 2 h post‐induction or the 417 IE genes. %, proportion of genes containing the indicated motifs. The CGyG core binding sequence of calmodulin‐binding transcription activator (CAMTA) transcription factors is highlighted in blue. FE, fold enrichment relative to the frequency in the complete genome. FDR, enrichment false discovery rate. The references for the motifs shown here are as follows: (1–4, 7) Finkler *et al*. (2007); (5) Nover *et al*. (1996); (6) Pandey & Somssich (2009), Weirauch *et al*. (2014); (9) Jaspar (2018) database motif profile MA0559.1. (e) CAMTA and CAMTA‐like motifs in proximal regions of the transcription start sites of IE genes and all Arabidopsis genes. TSS, transcription start site.

A distinct set of genes that respond immediately to a stimulus, such as a pathogen‐derived elicitor, are called ‘immediate‐early (IE) genes’ (Pauw & Memelink, 2004). The responsiveness of such early response genes is not influenced by treatment with a eukaryotic protein synthesis inhibitor, such as cycloheximide (CHX), as their regulation does not rely on *de novo* protein synthesis. A comparison of the transcriptome profile obtained on expression of MLA_CC_ at 2 h post‐induction with two independently conducted experiments examining CHX‐induced transcriptional profiles in seedlings (William *et al*., 2004; Goda *et al*., 2008) revealed that *c*. 87% of the MLA_CC_‐induced genes (417/478 genes) were upregulated in response to CHX treatment (log_2_FC > 1 and FDR < 0.05, Fig. 3a). Remarkably, the majority of MLA_CC_‐ and CHX‐induced genes were also upregulated on conditional activation of RPS4 at 2 h post‐induction, on application of flg22 at 0.5 h in seedlings (Fig. 3b) and in several other ETI and PTI responses (Fig. S4). Taken together, the observed responses suggest that these genes are common IE targets of ETI and PTI. Furthermore, the observed up‐regulation in response to CHX suggests that short‐lived repressors, which are constantly synthesized under resting conditions, might negatively regulate the expression of this set of genes.

Time‐resolved expression profiles of the aforementioned 417 genes during various ETI responses revealed that these genes were strongly induced during incompatible interactions, whereas their induction was less prominent in a compatible host–pathogen interaction (Fig. 3c), suggesting that they might be repressed by pathogen effectors. As these 417 genes are also induced in the absence of pathogens following conditional activation of MLA_CC_‐ and RPS4‐mediated responses (Fig. 3b,c), their transcriptional up‐regulation observed during ETI can be PAMP independent. Given that the early transcriptional response triggered by MLA_CC_ is similar to several abiotic stress responses (Fig. 2), at least part of the IE genes appear to be convergent targets in early stress signalling, and the mechanism underlying the transcriptional regulation of these IE genes might be conserved in biotic and abiotic stress responses. Hereafter, these 417 primary response genes are called IE genes (Table S2). The expression pattern of seven IE genes was validated by reverse transcription‐quantitative polymerase chain reaction (RT‐qPCR) analysis on expression of a series of MLA variants/truncated forms, and it was found that the full induction of these genes required a functional P‐loop in the full‐length MLA protein and dimerization of the MLA_CC_ domain (Fig. S1f).

To functionally categorize the 417 IE genes, we performed a gene ontology (GO) term enrichment analysis. This analysis indicated that the IE gene set was enriched, amongst other terms, for genes linked to biological processes in responses to various stimuli, including immune response, cell death and signal transduction (Table S3). Accordingly, there was an enrichment observed for genes involved in signal transduction and associated with kinase activity, catalytic activity or receptor activity (Table S3). There was also a significant enrichment for genes associated with the cellular component GO term membrane (Table S3). Taken together, this analysis shows that the identified IE genes contain a disproportionately high number of genes known to be involved in the perception and transduction of various biotic and abiotic stimuli.

Next, we investigated the regulatory mechanism(s) involved in the control of IE genes by identifying *cis*‐regulatory elements in their 5′ regulatory regions. An analysis based on the 5′ regulatory regions of all 562 genes induced by MLA_CC_ at 2 h post‐induction identified nine enriched sequence motifs using several independent methods (Fig. 3d). The same motifs were also found to be enriched in the 5′ regulatory regions of the 417 IE genes (Fig. 3d), indicating that these motifs are highly correlated with the IE response. The two most enriched motifs have been described previously as either binding motifs for CAMTAs (Bouché *et al*., 2002; Finkler *et al*., 2007) or as ABA‐responsive elements (ABRE‐CE; Hobo *et al*., 1999) (Fig. 3d). Notably, ABRE‐CE encompasses the CGyG sequence (Fig. 3d), which is the core‐binding motif of CAMTAs (Finkler *et al*., 2007). CAMTA‐binding motifs and CAMTA‐related motifs are present in up to 68% of the 417 IE genes, suggesting that CAMTA family proteins play a prominent role in the early transcriptional immune response.

Our *in silico* enrichment analysis of *cis*‐acting elements is experimentally strongly supported by the recently described Arabidopsis cistrome dataset (O'Malley *et al*., 2016), in which two CAMTA proteins (CAMTA1 and CAMTA5) preferentially bind CAMTA‐ and CAMTA‐related motifs in the regulatory regions of the IE genes (Table S4). In this dataset, CAMTA1‐ and CAMTA5‐targeted genes correspond to 5.5% and 1.6% of Arabidopsis genes, respectively, whereas the proportion of CAMTA1‐ and CAMTA5‐targeted IE genes reaches 29% and 9.3%, respectively (Table S4). The binding of two ABA‐responsive element‐binding proteins (AREB1 and AREB3) on IE genes is also significantly higher, but less pronounced, relative to that of the CAMTA proteins (Table S4). These findings prompted us to further study the role of the CAMTA family in early innate immune responses.

We examined the spatial distribution of the CAMTA and CAMTA‐related motifs around the transcription start site of the IE genes (Fig. 3e). These binding motifs are mainly located 0–300 bp upstream of the transcription start site of the IE genes (Fig. 3d,e). This pattern is consistent with the majority of transcription factor binding sites being located in proximal regions of the transcription start site in *A. thaliana* and *Arabidopsis* relatives (Yu *et al*., 2016). Taken together, these data suggest that these motifs are biologically relevant for IE gene expression.

### CAMTA family proteins modulate the primary transcriptional response in PTI and CNL‐triggered ETI

Previous studies have demonstrated that higher steady‐state levels of CAMTA3 negatively correlate with disease resistance (Jing *et al*., 2011; Zhang *et al*., 2014), and that CAMTA3 undergoes proteasome‐mediated degradation during ETI (Zhang *et al*., 2014). To build on these findings, we first examined CAMTA3 protein levels during PTI using a transgenic line in which CAMTA3‐FLAG complements the *camta3* knockout mutation (Du *et al*., 2009). We detected a weak and transient reduction in CAMTA3‐FLAG steady‐state levels on infiltration of flg22 at 1 hpi (Fig. S5). This suggests that degradation of CAMTA3 is common to PTI and ETI.

Similar to transgenic plants overexpressing CAMTA3, a dominant‐interfering CAMTA3 mutant variant carrying an A855V substitution exhibits severe defects in disease resistance, including ETI and systemic acquired resistance (SAR) (Jing *et al*., 2011; Nie *et al*., 2012). This dominant mutant (hereafter called *camta3‐D*) can be used to assess the function of CAMTA family members irrespective of their presumed functional redundancy (Kim *et al*., 2013), whilst overcoming the potential confounding effect of NLR activation observed in the major *camta* loss‐of‐function mutants (Lolle *et al*., 2017). To examine whether CAMTA family proteins are involved in the early transcriptional response shared by PTI and ETI, we generated RNA‐seq data to compare flg22‐ and RPM1‐mediated early transcriptional responses between wild‐type and *camta3‐D* plants.

**Figure 4:**
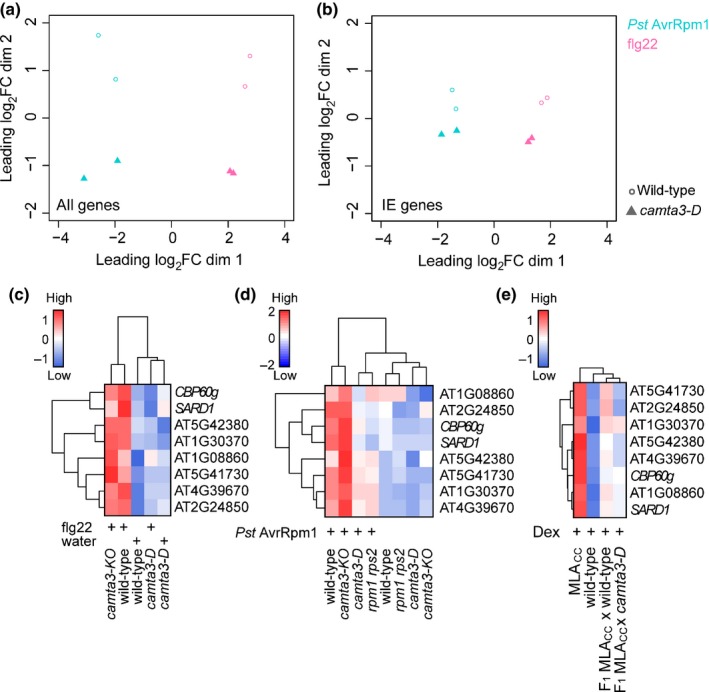
A dominant *camta3* mutation compromises pattern‐triggered immunity (PTI)‐ and effector‐triggered immunity (ETI)‐mediated transcriptional responses in *Arabidopsis thaliana*. (a, b) Multidimensional scaling (MDS) plots visualizing the similarity of gene expression profiles between samples considering (a) the complete set of Arabidopsis genes or (b) the 417 immediate‐early (IE) genes. RNA samples were prepared at 1 and 4 h post‐infiltration with flg22 and *Pseudomonas syringae* pv. *tomato* (*Pst*) DC3000 expressing AvrRpm1, respectively. MDS plots were created from trimmed mean of M‐values (TMM)‐normalized log_2_ counts per million, using the pairwise Euclidean (root‐mean‐square deviation) distance between samples as distance measure. Thus, distances in the plot can be interpreted as approximations of the typical expression log_2_ fold changes between samples. RNA‐seq data for this analysis were obtained from two independent experiments. (c) At 1 h post‐infiltration with flg22 (or water), transcript levels of IE genes are significantly higher in flg22‐treated wild‐type plants than in the other conditions (*P *<* *0.05). (d) At 4 h post‐infiltration with *Pst* DC3000 expressing AvrRpm1, transcript levels of IE genes in infected plants are significantly higher in wild‐type than in *camta3‐D* plants (*P *<* *0.05). (e) On conditional expression of the Mildew resistance locus A (MLA) coiled‐coiled domain (MLA_CC_), transcript levels of IE genes are significantly higher in wild‐type plants than in plants carrying the *camta3‐D* mutation (*P *<* *0.05). The expression of IE genes was analysed in F_1_ plants from a cross between a homozygous MLA_CC_ line and either wild‐type plants or *camta3‐D* mutants at 4 h post‐induction of MLA_CC_ by dexamethasone (Dex) infiltration. (c–e) Transcript levels of the six validated IE genes (see Supporting Information Fig. S1f) and two further positive defence regulators, *SARD1* (*AT1G73805*; IE gene) and *CBP60g* (*AT5G26920*), were examined by reverse transcription‐quantitative polymerase chain reaction (RT‐qPCR). The average from two independent replicates was used for visualization and statistical analysis. Expression patterns were grouped by hierarchical clustering (complete linkage). (a–e) *camta3‐D* and *camta3‐KO* indicate a dominant *camta3* mutant carrying an A855V substitution and a knockout *camta3* mutant, respectively.

To compare patterns of gene expression between samples, we applied multidimensional scaling (MDS) to the 500 genes with the largest fold changes in each pairwise comparison. We found that the early transcriptional response is clearly different between *camta3‐D* mutant and wild‐type plants in both treatments (Fig. 4a, along dimension 2). Similarly, the IE gene transcriptional response mediated by flg22 and RPM1 shows a separation between *camta3‐D* and wild‐type samples (Fig. 4b, along dimension 2). These data suggest that the CAMTA family is involved in early transcriptional reprogramming in PTI and ETI. To further study the observed impact of the *camta3‐D* mutation on IE gene expression (Fig. 4b), we determined the transcript levels of a subset of IE genes (*AT1G08860*,* AT1G30370*,* AT2G24850*,* AT4G39670*,* AT5G41730*,* AT5G42380*) by RT‐qPCR in wild‐type plants, *camta3‐D* and *camta3* knockout plants (*camta3‐KO*) during ETI and PTI (Fig. 4c–e). In addition to the IE genes, *CBP60g* (*Cam‐Binding Protein 60‐like G*) was included in this analysis as *CBP60g* plays a pivotal role in immunity, together with *SARD1* (*SAR Deficient 1*), one of the identified IE genes (Wang *et al*., 2011; Sun *et al*., 2015). We detected comparable IE gene expression patterns between wild‐type and *camta3‐KO* in both flg22‐ and RPM1‐mediated responses (Fig. 4c,d). Consistent with the RNA‐seq analysis, expression of these IE genes was significantly lower in *camta3‐D* relative to wild‐type plants (Fig. 4c,d). Similarly, MLA_CC_‐dependent IE gene expression in P‐FC was reduced in a *camta3‐D* background (Figs 4e, S1g). Collectively, these results suggest that CAMTA regulation of IE gene expression is integral to the studied ETI and PTI responses.

**Figure 5:**
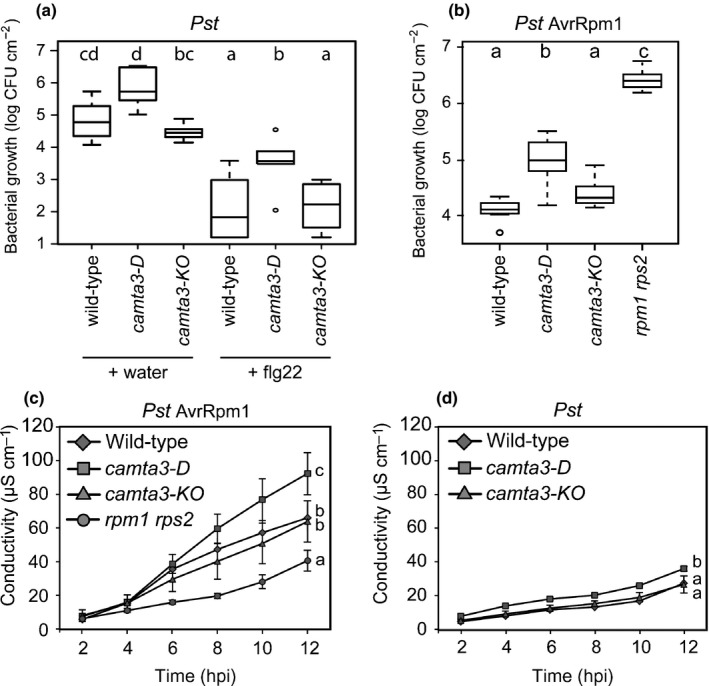
A dominant *camta3* mutation compromises flg22‐ and RPM1‐mediated disease resistance, but enhances host cell death response on pathogen challenges in *Arabidopsis thaliana*. (a) Bacterial growth at 2 d after infiltration of *Pseudomonas syringae* pv. *tomato* (*Pst*) DC3000 (OD_600_ = 0.0001). flg22 (1 μM) or water was infiltrated 24 h before *Pst* infiltration. (b) Bacterial growth at 3 d after infiltration of *Pst* expressing AvrRpm1 (OD_600_ = 0.0001). (a, b) Boxplots summarize the observed log_10_‐transformed bacterial counts. Different letters indicate significant differences (*P *<* *0.05; ANOVA with Tukey *post‐hoc* tests). CFU, colony‐forming unit; OD, optical density. Two independent experiments with two (a, b for *camta3‐KO* with *Pst* AvrRpm1) or three replicates per experiment were conducted. (c, d) Ion leakage assays on bacterial pathogen challenge. Samples were collected at 0.5 h post‐infiltration (hpi) with *Pst* (OD_600_ = 0.05) expressing (c) or lacking (d) AvrRpm1. Data were obtained in three independent experiments (*n *=* *5) for the avirulent and two independent experiments (*n *=* *4) for the virulent pathogen challenge. Means ± SE are shown. Different letters next to the lines indicate significant differences in ion leakage between genotypes (*P *<* *0.05; ANOVA with Tukey *post‐hoc* tests; data at 2 and 4 hpi are excluded). (a–d) *camta3‐D* and *camta3‐KO* indicate a dominant *camta3* mutant carrying an A855V substitution and a knockout *camta3* mutant, respectively.

Pathogen growth suppression on flg22 pretreatment or by activated RPM1 was compromised in *camta3‐D* plants, confirming and extending the relevance of the CAMTA family in PRR‐ and NLR‐mediated disease resistance (Fig. 5a,b) (Nie *et al*., 2012). The *camta3‐KO* mutant exhibited wild‐type‐like disease resistance against *Pst* after flg22 infiltration and *Pst* AvrRpm1 (Fig. 5a,b), probably as a result of functional redundancy within the CAMTA family (Kim *et al*., 2013). These data imply that CAMTA‐mediated primary transcriptional reprogramming associates with the disease resistance responses mediated by flg22 and RPM1.

ETI is often accompanied by a host localized cell death response (Maekawa *et al*., 2011b). To examine whether host cell death is altered in *camta3‐D* in RPM1‐triggered ETI, we quantified ion leakage from pathogen‐challenged leaves (Fig. 5c,d). Notably, the RPM1‐dependent cell death response was significantly enhanced in *camta3‐D* relative to wild‐type and *camta3‐KO* (Fig. 5c), even though *camta3‐D* plants are more disease susceptible (Fig. 5b). These data indicate that RPM1 becomes active in response to *Pst* DC3000 expressing AvrRpm1, but fails to mount an effective immune response in *camta3‐D*. Slightly increased ion release was also detected on inoculation of *camta3‐D* with virulent *Pst* (Fig. 5d). Collectively, these data suggest that the magnitude of the cell death response is inversely coupled to the effectiveness of RPM1‐mediated disease resistance, in which the CAMTA family might contribute to cell death suppression.

## Discussion

Here, we have demonstrated extensive similarity between PTI and ETI responses at the transcriptome level (Fig. 1), building on the findings from previous analyses (Eulgem *et al*., 2004; Navarro *et al*., 2004). Consistently, our time‐resolved comparative transcriptome analysis did not identify a marker gene that was specific to PTI or ETI at early time points. Further comparisons with transcriptional changes on abiotic stress responses showed that a part of the early transcriptional reprogramming is shared between biotic and abiotic stresses (Fig. 2), as described previously (Zou *et al*., 2011; Gu *et al*., 2016). This mode of rapid and transient gene regulation is referred to as a general stress response (GSR; Bjornson *et al*., 2014) and primes the plant for subsequent stress‐specific reactions (Walley & Dehesh, 2010; Bjornson *et al*., 2016). Our findings suggest that early ETI and PTI responses converge onto a common transcriptional output, which overlaps with the GSR. Consistent with this, enhanced expression of the Arabidopsis CNL ADR1 confers drought tolerance (Chini *et al*., 2004). In addition, adaptation to one stress condition can confer tolerance to other non‐related stresses (Tippmann *et al*., 2006; Perez & Brown, 2014). It is conceivable that this ‘cross‐tolerance’ (Tippmann *et al*., 2006) is in part a result of an overlap in the responding gene sets which are enriched in signal‐transducing components (Table S3; Fig. 2). Using a dominant *camta3* variant, we revealed an unexpected link between the CAMTA‐mediated early gene induction and cell death suppression (Fig. 5c,d). Thus, one of the functions of the GSR might be to protect cells from adverse cell death. As localized host cell death at sites of attempted pathogen invasion is often associated with ETI, many ETI responses appear to be able to overcome such anti‐cell death or pro‐survival activities. It is possible that the nuclear pore‐mediated signalling mechanism contributes to ETI‐associated cell death activation (Gu *et al*., 2016). Of note, the regulatory mechanism behind the early gene induction is uncoupled from those promoting host cell death, as PTI‐ and RPS4‐mediated responses in Arabidopsis accession Col‐0 are not associated with strong host cell death (Heidrich *et al*., 2013). Several lines of evidence suggest that effective disease resistance, including pretreatment with PAMPs, reduces the magnitude of NLR‐triggered cell death (Fig. 5; Rate & Greenberg, 2001; Hofius *et al*., 2009; Hatsugai *et al*., 2017). Such a mechanism would be advantageous for plants to minimize cellular damage during ETI.

An unresolved question in ETI‐associated transcriptional reprogramming is whether ETI amplifies or sustains PTI‐mediated transcriptional signatures on challenge with avirulent pathogens (Caldo *et al*., 2004; Day & He, 2010; Maekawa *et al*., 2012; Garcia & Hirt, 2014; Vidhyasekaran, 2014; Cui *et al*., 2015). As plant interactions with avirulent pathogens invariably result in the co‐activation of PTI and ETI, we studied here responses induced by P‐FC activation of the TNL RPS4 or the MLA_CC_ domain, or an autoactive full‐length version of the CNL MLA. Conditional NLR activation was sufficient to induce transcriptional changes that were highly similar to those observed in pathogen‐triggered ETI responses (Fig. 1a). This finding indicates that transcriptional regulation in ETI can be established independently of PTI. Of note, we established here that the barley MLA_CC_ domain induces both cell death and immune transcriptional reprogramming in stable transgenic lines of a heterologous dicot species. As many pathogen effectors target signalling components of PTI (Block & Alfano, 2011; Xin & He, 2013; Macho & Zipfel, 2015), the ETI and cell death machinery appears to be resilient against pathogen effector‐mediated interception of PTI signalling.

In plant and animal innate immunity, the majority of rapidly induced genes responding to a microbial stimulus appear to be primary response genes whose regulation is independent of *de novo* protein synthesis (Pauw & Memelink, 2004; Smale, 2012). By comparing plant transcriptional responses to conditional MLA_CC_ expression and CHX treatments, we defined a set of IE genes, most of which are rapidly induced in PTI and ETI (Figs 3, S3; Table S2). In animal immunity, although some primary response genes encode cytokines, chemokines and molecules that directly limit pathogen growth, the majority encode signalling components, such as transcription factors, contributing to the activation of secondary responses in the transcriptional cascade (Smale, 2012). GO term enrichment analysis showed that the plant IE genes encode a disproportionally high number of proteins involved in signal perception and transduction (Table S3), similar to the animal immediate early immune response (Smale, 2012).

The analysis of promoter sequences of the primary response genes shared in NLR‐ and PRR‐mediated signalling implicated the CAMTA protein family as a major regulator for these genes (Fig. 3d). CAMTA3 has been proposed as a negative regulator of plant immunity, based mainly on elevated immunity in *camta3* knockout mutants (i.e. *camta3‐KO*) (Du *et al*., 2009; Nie *et al*., 2012; Zhang *et al*., 2014). However, a recent study has demonstrated that the autoimmunity of *camta3* knockout plants is mainly a result of the ectopic activation of two NLRs (Lolle *et al*., 2017). Thus, it remains unclear whether CAMTA3 and other CAMTA members act as positive (Choi *et al*., 2005; Doherty *et al*., 2009; Benn *et al*., 2014) or negative (Du *et al*., 2009) regulators of transcription. In our experiments, we were unable to detect a difference in the expression of selected IE genes between wild‐type and *camta3‐KO* plants during PTI and ETI (Fig. 4). At 19–21°C, *camta3‐KO* plants exhibited a plant age‐associated retarded growth accompanied by enhanced disease resistance (Du *et al*., 2009). It is possible that, under our conditions (with higher temperature: 21–22°C; see the Materials and Methods section), growth and gene expression of *camta3‐KO* mutants remained comparable with wild‐type plants. Using a dominant mutant of CAMTA3 (*camta3‐D*), we provide genetic evidence linking CAMTA3 and/or other CAMTA members to transcriptional regulation during flg22‐mediated PTI and CNL‐mediated ETI (Fig. 4). The molecular mechanism underlying the effect of the dominant interfering *camta3‐D* mutation on defence outputs remains to be determined. A recent study (Kim *et al*., 2017) has shown that the CAMTA3 N‐terminal domain alone is able to repress SA‐related gene induction in response to low temperature in *camta2 camta3* plants. Considering that CAMTA3 is a downstream target of PTI and ETI as shown here, CAMTA3‐D protein still binds to CaM (Nie *et al*., 2012) and the *CAMTA3‐D* mutation is dominant over the loss of CaM‐binding mutation (Kim *et al*., 2017), we propose that a CAMTA3‐D N‐terminal domain repressor function is active irrespective of CaM‐binding status.

Given the observed regulatory function of CAMTAs in both PTI and ETI, CAMTAs might be targeted by pathogen effectors to promote pathogen virulence. Consistent with this idea, the loss of *CAMTA3* activates NLRs, which results in host cell death (Lolle *et al*., 2017). Hence, several NLRs are able to reboot resistance, possibly via host cell death activation, even when effectors disable the CAMTA‐mediated transcriptional machinery (Lolle *et al*., 2017). Monitoring of CAMTA activity by NLRs further underlines the importance of this protein family in plant innate immunity. NLR‐mediated surveillance of CAMTA activity could be a mechanism of the plant innate immune system to ensure resilience of convergence points in ETI and PTI against manipulation by pathogen effectors.

## Author contributions

F.J., P.S‐L. and T.M. designed the research; F.J. and T.M. performed the research; A.M., C.S., S.B‐B., J.E.P. and K.T. provided the data; F.J., B.K. and T.M. analysed the data; F.J., B.K., P.S‐L. and T.M. wrote the paper with co‐author contributions.

## Supporting information

Please note: Wiley Blackwell are not responsible for the content or functionality of any Supporting Information supplied by the authors. Any queries (other than missing material) should be directed to the *New Phytologist* Central Office.

**Fig. S1** Induction of cell death and growth defects on expression of MLA (Mildew resistance locus A) variants/truncated forms in *Arabidopsis thaliana* leaves.
**Fig. S2** Predominance of gene induction over repression in the early immune response.
**Fig. S3** Expression profile of rapidly MLA_CC_ (Mildew resistance locus A coiled‐coil)‐responsive genes during various early immune‐related, abiotic stress‐induced, phytohormone‐induced and chemically induced responses.
**Fig. S4** Induction of immediate‐early (IE) genes in selected effector‐triggered immunity (ETI) and pattern‐triggered immunity (PTI) responses compared with conditional MLA_CC_ (Mildew resistance locus A coiled‐coil) expression.
**Fig. S5** Treatment with the pathogen‐associated molecular pattern (PAMP) flg22 leads to a rapid and transient decrease in calmodulin‐binding transcription activator 3 (CAMTA3) protein steady‐state level.
**Table S1** Summary of the transcriptomic datasets used in this study
**Table S2** Expression data (log_2_ fold change (log_2_FC)) of the 478 genes induced by MLA_CC_ (Mildew resistance locus A coiled‐coil) at 2 h post‐induction during the early response to various stressors
**Table S3** Gene ontology (GO) term enrichment analysis of the 417 immediate‐early (IE) genes induced by MLA_CC_ (Mildew resistance locus A coiled‐coil) at 2 h post‐induction
**Table S4** Cistrome data for calmodulin‐binding transcription activator (CAMTA) proteins and abscisic acid (ABA)‐responsive element‐binding proteins
**Methods S1** Methods related to RNA‐seq data acquisition including pathogen inoculation and transcriptomic analysis.
