# Supplementary material for: A dominant‐interfering camta3 mutation compromises primary transcriptional outputs mediated by both cell surface and intracellular immune receptors in Arabidopsis thaliana
Source: New Phytol. 2017 Dec 11;217(4):1667–80. doi: 10.1111/nph.14943 (PMC5873390; doi:10.1111/nph.14943)
Supplement: Supplementary file 1 — Fig. S1 Induction of cell death and growth defects on expression of MLA (Mildew resistance locus A) variants/truncated forms in Arabidopsis thaliana leaves. Fig. S2 Predominance of gene induction over repression in the early immune response. Fig. S3 Expression profile of rapidly MLACC (Mildew resistance locus A coiled‐coil)‐responsive genes during various early immune‐related, abiotic stress‐induced, phytohormone‐induced and chemically induced responses. Fig. S4 Induction of immediate‐early (IE) genes in selected effector‐triggered immunity (ETI) and pattern‐triggered immunity (PTI) responses compared with conditional MLACC (Mildew resistance locus A coiled‐coil) expression. Fig. S5 Treatment with the pathogen‐associated molecular pattern (PAMP) flg22 leads to a rapid and transient decrease in calmodulin‐binding transcription activator 3 (CAMTA3) protein steady‐state level. Table S1 Summary of the transcriptomic datasets used in this study Table S2 Expression data (log2 fold change (log2FC)) of the 478 genes induced by MLACC (Mildew resistance locus A coiled‐coil) at 2 h post‐induction during the early response to various stressors Table S3 Gene ontology (GO) term enrichment analysis of the 417 immediate‐early (IE) genes induced by MLACC (Mildew resistance locus A coiled‐coil) at 2 h post‐induction Table S4 Cistrome data for calmodulin‐binding transcription activator (CAMTA) proteins and abscisic acid (ABA)‐responsive element‐binding proteins Methods S1 Methods related to RNA‐seq data acquisition including pathogen inoculation and transcriptomic analysis. [file NPH-217-1667-s001.pdf]

***New Phytologist* Supporting Information**

Article title: A dominant-interfering *camta3* mutation compromises primary transcriptional outputs mediated by both cell surface and intracellular immune receptors in *Arabidopsis thaliana*

Authors: Florence Jacob, Barbara Kracher, Akira Mine, Carolin Seyfferth, Servane Blanvillain-Baufumé, Jane E. Parker, Kenichi Tsuda, Paul Schulze-Lefert and Takaki Maekawa

Article acceptance date: 08 November 2017

The following Supporting Information is available for this article:

Fig. S1 Induction of cell death and growth defects upon expression of MLA variants/truncated forms in *Arabidopsis thaliana* leaves.

Fig. S2 Predominance of gene induction over repression in the early immune response.

Fig. S3 Expression profile of rapidly MLA_{CC}-responsive genes during various early immune-related, abiotic stress-, phytohormone- and chemically-induced responses.

Fig. S4 Induction of immediate-early (IE) genes in selected ETI and PTI responses compared to conditional MLA_{CC} expression.

Fig. S5 Treatment with the PAMP flg22 leads to a rapid and transient decrease of CAMTA3 protein steady state level.

Table S1 Summary of the transcriptomic datasets used in this study. [submitted as a separate file]

Table S2 Expression data (log_2FC) of the 478 genes induced by MLA_{CC} at 2 hours post induction during the early response to various stressors. [submitted as a separate file]

Table S3 Gene ontology (GO) term enrichment analysis of the 417 IE genes induced by MLA_{CC} at 2 hours post induction. [submitted as a separate file]

Table S4 Cistrome data for CAMTA proteins and ABA-responsive element-binding proteins [submitted as a separate file]

Methods S1 Methods related to RNA-seq data acquisition including pathogen inoculation and transcriptomic analysis

References related to Supporting Information

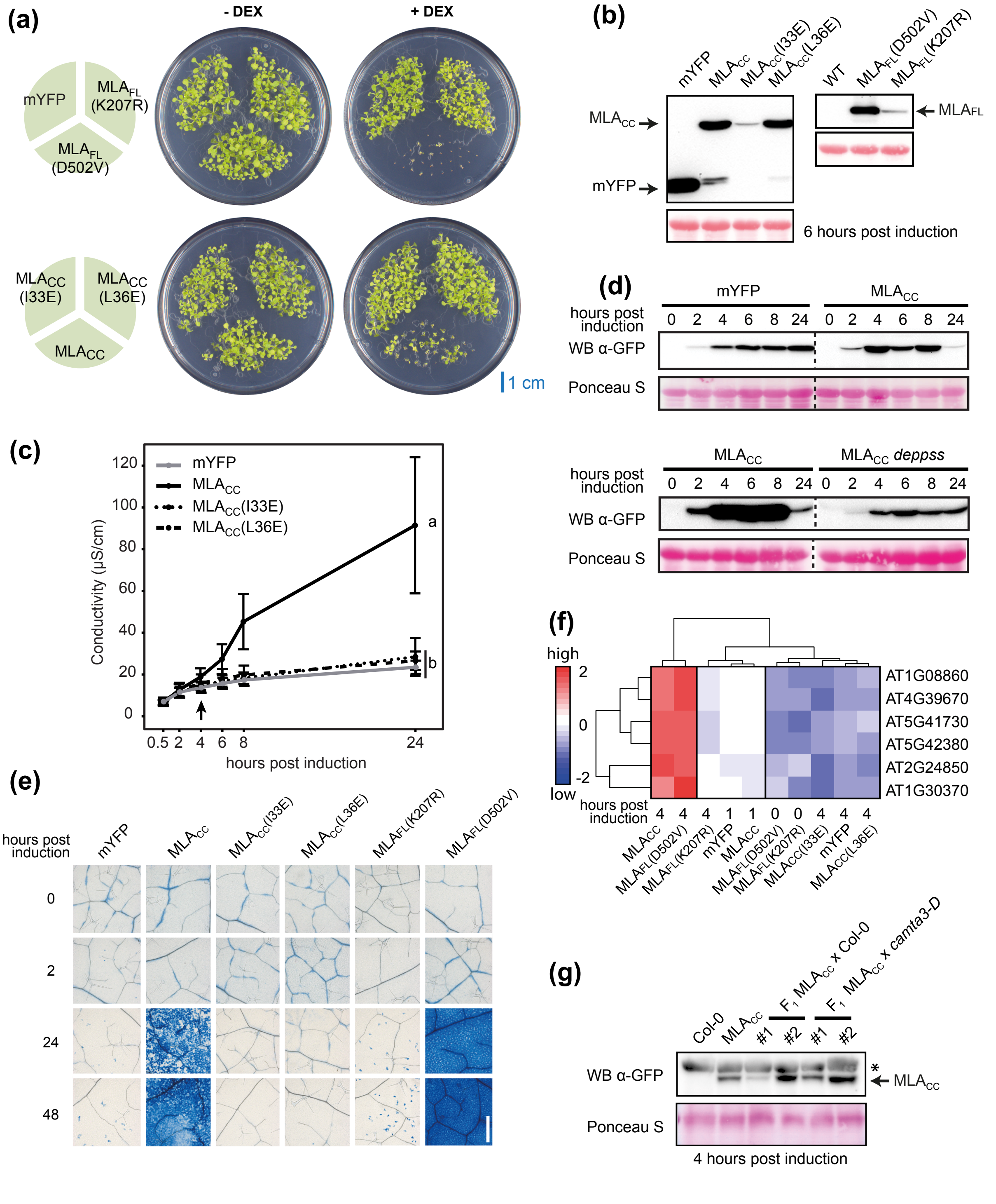

Fig. S1 Induction of cell death and growth defects upon expression of MLA variants/truncated forms in *Arabidopsis thaliana* leaves. Arabidopsis stable transgenic lines that conditionally express the MLA coiled-coil domain (MLA$_{CC}$), two variants lacking the ability of homodimerization (MLA$_{CC}$(I33E), MLA$_{CC}$(L36E)), or MLA full length protein carrying an autoactive mutation (MLA$_{FL}$(D502V)), or a loss of function mutation (MLA$_{FL}$(K207R)) were used.

A plant line that conditionally expresses monomeric YFP (mYFP) was used as a negative control. MLA_{CC} variants are C-terminally fused with mYFP and MLA_{FL} variants are C-terminally fused with 3xTY1 epitope tags. **(a)** DEX-inducible expression of the autoactive MLA variants MLA_{FL}(D502V) and MLA_{CC} leads to growth retardation in Arabidopsis stable transgenic lines. The transgenic lines used in this assay are indicated in the left panels. The pictures were taken at 16 days after sowing on medium without DEX (- DEX) or with 10 µM DEX (+ DEX). **(b)**-**(g)** Samples were collected from leaves of four-week-old plants after infiltration of 1 µM DEX at the indicated time points. **(b)** Steady state levels of the MLA variants/truncated forms after DEX infiltration. Samples were examined by immunoblotting anti-GFP or anti-TY1. Ponceau S staining was used to monitor equal loading. **(c)** Quantification of cell death by ion leakage measurement. The data represent the mean and standard deviation of at least nine biological replicates obtained from at least three independent experiments with at least three biological replicates each. Statistical differences between the genotypes were assessed using ANOVA followed by Tukey HSD (P<0.05). The arrows indicate the earliest time point where statistical differences between the autoactive and inactive variants can be detected. **(d)** Temporal analysis of protein steady state levels by immunoblotting with anti-GFP antibodies in the lines expressing MLA_{CC}-mYFP and mYFP under a DEX-inducible promoter. Ponceau S staining was used to monitor equal loading. **(e)** Trypan blue staining of leaves. The adaxial surface of leaves was imaged by bright field microscopy. Most of the stained cells are localized in the mesophyll layer. Scale bar: 500 µm. **(f)** Transcript levels of the selected IE genes were measured by RT-qPCR with at least three independent experiments and means of standardized expression levels are shown. Hierarchical clustering (complete linkage) indicates three major expression patterns. The gene-wise transcript levels are significantly higher in MLA_{CC} and MLA_{FL}(D502V) lines at 4 hours post induction compared to the other conditions (P<0.05). The gene-wise transcript levels in MLA_{FL}(K207R) are significantly higher than in MLA_{CC}(I33E), MLA_{CC}(L36E), and mYFP at 4 at 4 hours post induction suggesting a residual activity of this variant to induce the IE genes (P<0.05). **(g)** MLA_{CC}-mYFP protein steady state levels in *camta3-D* and control plants at 4 at 4 hours post induction. The homozygous line carrying the expression construct was crossed with either wild-type plants or *camta3-D* mutants and their siblings (F_1) were used. *camta3-D* plants were used as female parents and the number at the panel indicates seed batches derived from different male parents.

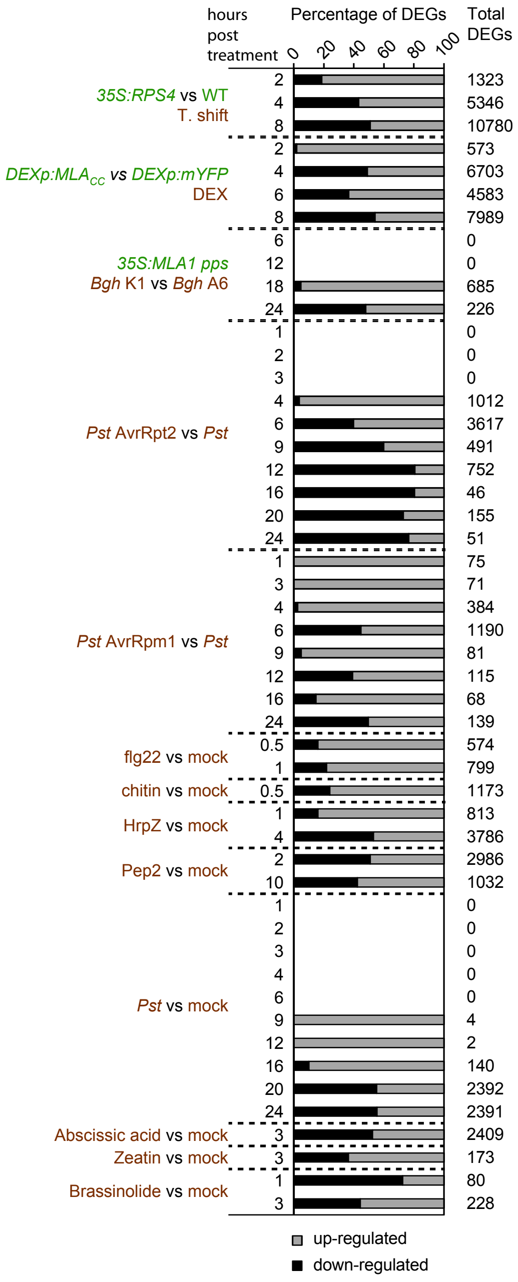

Fig. S2 Predominance of gene induction over repression in the early immune response. The number and proportion of differentially expressed genes (DEGs) are shown during various immune-related or non-immune related responses. Unless otherwise specified, the genotype is wild-type Arabidopsis (Col-0). Genotypes different from Col-0 are indicated in green, and the applied treatments are indicated in brown. The response to *Pst* AvrRps4 was not included in this visualization since no DEGs were identified under our settings. DEX: dexamethasone. T. shift: temperature shift.

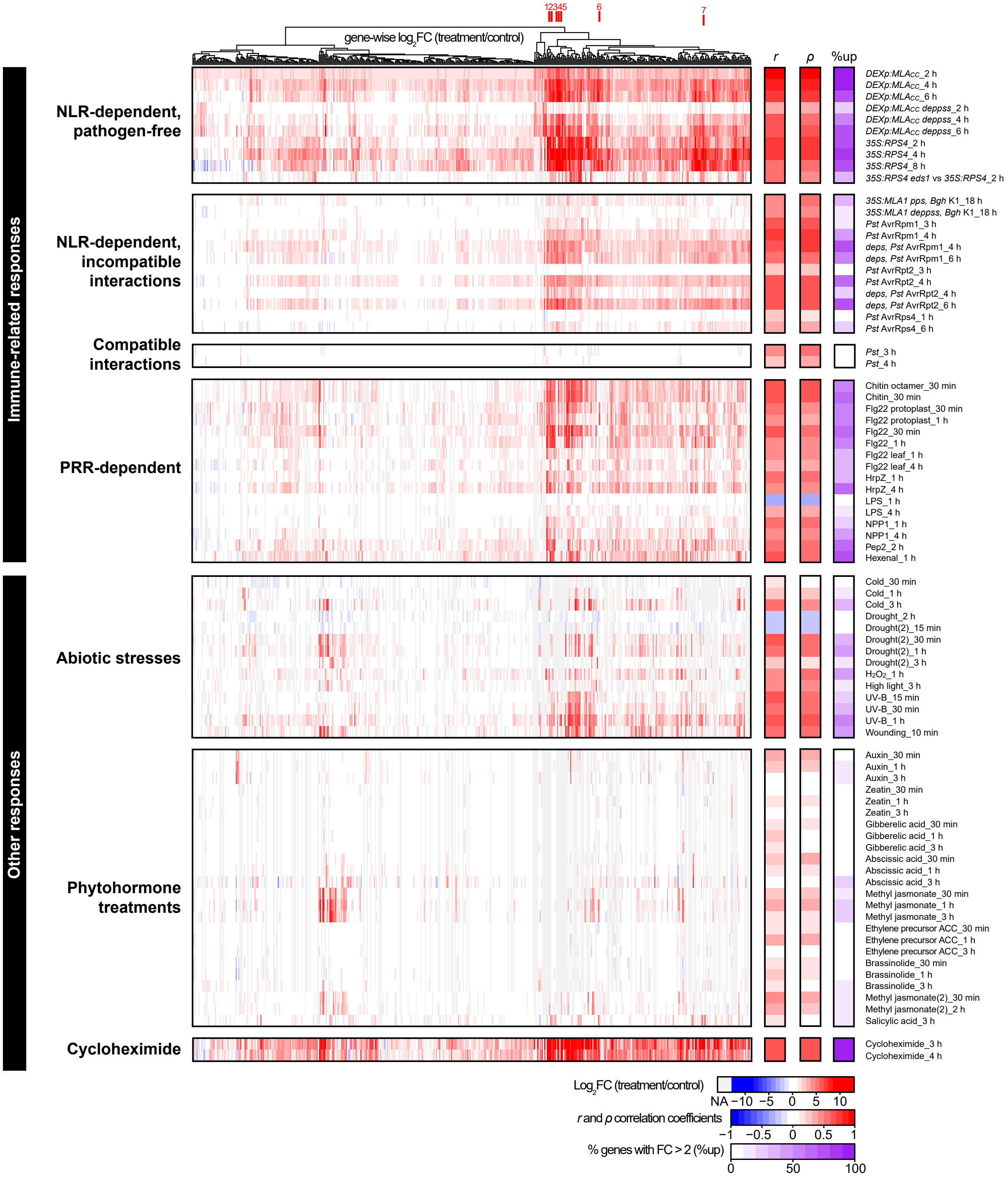

Fig. S3 Expression profile of rapidly MLA_{CC}-responsive genes during various early immune-related, abiotic stress-, phytohormone- and chemically-induced responses. The analysis is based on a cluster of 478 genes, which was extracted from the 562 genes induced by conditional MLA_{CC} expression at 2 hours post induction. The figure shows from left to right: heatmap representation of the hierarchically-clustered gene-wise log_2FC (treatment/control) values; pairwise Pearson's and Spearman's correlation coefficients (*r* and *ρ* respectively) of the

log$_2$FC (treatment/control) values between each of the other treatments and the conditional MLA$_{CC}$ expression at 2 hours post induction; and percentage of genes in this cluster with more than 2-fold induction (%up) in the indicated condition. The position of the seven genes selected for RT-qPCR and induced upon MLA$_{CC}$ expression at 2 hours post induction is indicated by red ticks: *AT1G30370* (1), *AT4G39670* (2), *AT5G41730* (3), *AT1G08860* (4), *AT5G42380* (5), *AT2G24850* (6), and *SARD1* (7). A representative subset of the data presented here is shown in Fig. 2. Information for the datasets used in this study is provided in Table S1. NA, not available (expression not detected).

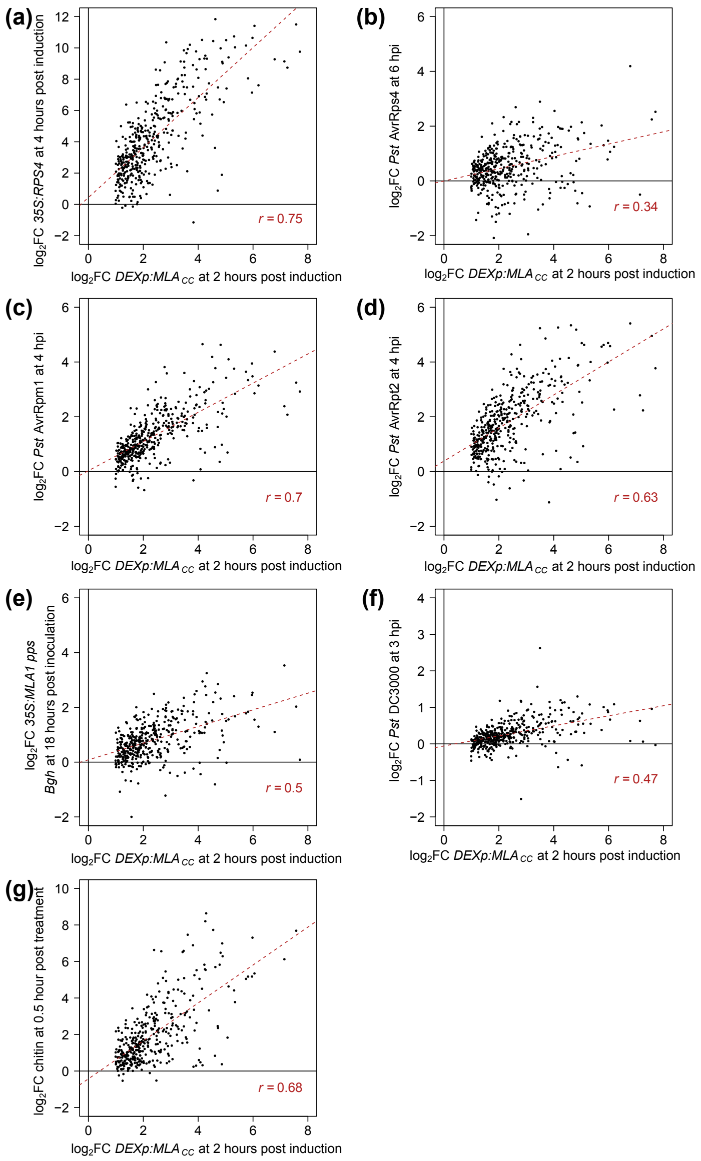

Fig. S4 Induction of immediate-early (IE) genes in selected ETI and PTI responses compared to conditional MLA$_{CC}$ expression. The scatter plots show the expression changes (log$_2$FC) of the 417 immediate-early (IE) genes upon MLA$_{CC}$ expression at 2 hours post induction in comparison to the changes induced **(a)** upon conditional activation of the RPS4-mediated response at 4 hours post induction, **(b)** upon infection with *Pseudomonas syringae* pv. *tomato* DC3000 (*Pst*)

expressing AvrRps4 at 6 hpi, **(c)** upon infection with *Pst* expressing AvrRpm1 at 4 hpi, **(d)** upon infection with *Pst* expressing AvrRpt2 at 4 hpi, **(e)** upon infection of plants expressing *35S:MLA1* with *Blumeria graminis* f. sp. *hordei* isolate K1 (*Bgh* K1) at 18 hours post inoculation, **(f)** upon infection with virulent *Pst* at 3 hpi, and **(g)** upon chitin treatment at 0.5 hours. The corresponding pairwise Pearson's correlation coefficients calculated based on the log_2FC (treatment/control) values of these 417 IE genes are indicated in each plot in dark red. hpi: hours post infiltration.

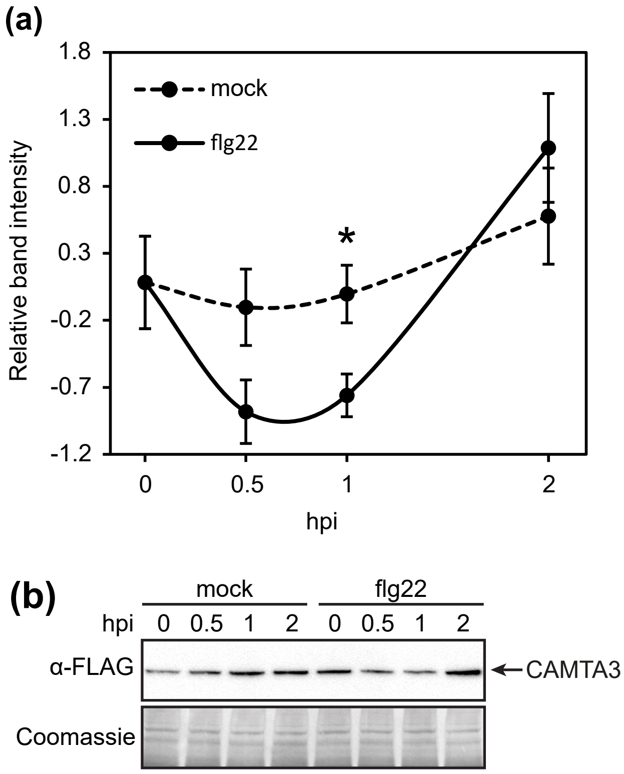

Fig. S5 Treatment with the PAMP flg22 leads to a rapid and transient decrease of CAMTA3 protein steady state level. (a) Semi-quantification of CAMTA3-FLAG steady state level by immunoblots. The mean and standard errors of standardized protein levels were obtained from six independent experiments (n=6). The difference between mock and flg22 treatment is statistically significant at 1 hpi ($P<0.05$, Welch Two Sample *t*-test, indicated by asterisk). **(b)** Representative pattern of immunoblot observed in three out of six independent experiments. Equal loading and blotting was monitored by Coomassie Brilliant Blue staining (lower panel). CAMTA3-FLAG protein abundance was analysed in stable transgenic complementation lines by immunoblot analysis using anti-FLAG antibodies following leaf-infiltration with water (mock) or 100 nM flg22. hpi: hours post infiltration.

Methods S1 Methods related to RNA-seq data acquisition including pathogen inoculation and transcriptomic analysis

RNA-seq

Leaf samples from at least five independent plants were collected at the indicated time point after treatment. Total RNA was extracted using the RNeasy Plant Mini Kit (Qiagen, Venlo, Netherlands). Total RNA was DNase-treated and purified using RNase-Free DNase Set (Qiagen) and the RNeasy MinElute kit (Qiagen). Ribosomal RNAs were depleted from 2.5 μg RNA using the RiboMinus Plant Kit (Thermo Fisher Scientific, Waltham, MA, USA) for RNA-seq. For the RNA-seq data generated in this study, mRNA sequencing libraries were prepared with barcoding using the TruSeq RNA Sample Preparation Kit (Illumina, San Diego, CA, USA). All procedures were performed according to the manufacturer's instructions. The barcoded libraries were pooled together and sequenced by Illumina HiSeq2000 or Illumina HiSeq2500, yielding between 18 and 40 Mio 100 bp reads per sample. Unless otherwise indicated, three independent biological replicates were processed and analysed for each experimental condition.

RNA-seq data analysis

Raw RNA-seq data were collected from public datasets or obtained in this study (Table S1). Total reads were mapped onto the *A. thaliana* genome (TAIR10) using TopHat2 (Kim *et al.*, 2013). Read counts per gene were calculated from the mapped RNA-seq reads using HTseq-count function, apart from our previously published *35S:MLA1 pps* dataset for which the CoverageBed (bedTools suite, Quinlan & Hall, 2010) had been used. Genes with less than 100 reads within one dataset were discarded, and the $\log_2$-transformed count data was normalized using the function voom from the R package limma (www.r-project.org, Smyth, 2005) resulting in $\log_2$ counts per million. Differential gene expression between genotypes and/or treatments and/or times, was analysed by fitting a linear model with the appropriate explanatory variables using the function lmFit (R package limma). Log_2-transformed fold change ($\log_2$FC) values between sample and control at the same time point were used for most of the comparative transcriptomic analysis conducted in this study. Statistical analysis was performed using moderated *t*-tests over the contrasts of interests and the resulting *p*-values were corrected for multiple testing using the Benjamini-Hochberg method. The criteria for significant differential expression were: FDR < 0.01 and $|\log_2\text{FC}| > 1$. MDS plots were created in R from the TMM normalized log2 counts per million using the function plotMDS (R package lima) with default parameter settings, i.e. for each pair of samples the distance is calculated from the top 500 genes that best distinguish these two samples (unless the analyzed gene set contains less than 500 genes, in which case all genes will be included).

Microarray data analysis

Publicly available experiments using the Affymetrix ATH1-121501 platform were obtained from several data sources (Table S1). Only experiments including at least three biological replicates were selected. The raw .cel files were downloaded and normalized with Robust Multi-array Average (RMA) normalization as implemented in BioConductor (Irizarry *et al.*, 2003; Gentleman

et al., 2004). Probe annotation was performed using the ath1121501ACCNUM from the ath1121501.db annotation data. Probes with no or ambiguous annotations were removed. For each dataset the log_2-base fold changes (log_2FC) of treatment versus control were computed by fitting a linear model with the appropriate explanatory variables using the function lmFit (R package limma). This log2FC was used for the comparative transcriptomic analysis described in this study. When necessary, differentially expressed genes were extracted using the R/Bioconductor package limma (Ritchie *et al.*, 2015) with the criteria $|log_2FC|>1$ and FDR<0.05.

Transcript quantification by RT-qPCR

Leaf samples from at least five independent plants were collected at the indicated time points after treatment. Total RNA was extracted with the RNeasy Plant Mini Kit (Qiagen) according to the manufacturer instructions. Reverse transcription was performed using the Quantitec Reverse Transcription kit (Qiagen) according to the manufacturer's instructions. The cDNA was subjected to qPCR with gene-specific primers using the IQ SYBR Green reagent (Bio-Rad, Hercules, CA) on the iQ5 Multicolor Real-Time PCR Detection System (Bio-Rad) with three technical replicates for each of the three biological replicates unless otherwise stated. The relative expression was normalized to *AT4G26410*, which was previously described as highly constant under varying stress conditions (Czechowski *et al.*, 2005). For validation of the RNA-seq experiment and further characterization of the CAMTA3-dependent gene regulation, we selected 6 genes among the 10 most induced IE genes at 2 hours post MLA_{CC} induction. *CBP60g* (*AT5G26920*) and *SARD1* (*AT1G73805*) were also included due to their known function in the transcriptional regulation of immune responses (Wang *et al.*, 2011; Sun *et al.*, 2015). The genes and their respective primer pairs (in 5 '-to- 3 ' direction) were as follows: *AT1G08860* (fw: GGTTCTGAAAGGCCAGCTCA, rv: TCGCCATTTGAAGCAGTGAAG), *AT1G30370* (fw: GTCGACGTTCCTCAGTGGTT, rv: AACCATTCCGTGGGAGTCAC), *AT1G73805* (fw: CTTGCCTCGCCAATTTCCAG, rv: ACGGAAAGACGATGACCGAG), *AT2G24850* (fw: ACGATCTTCTCCCCGAGAGT, rv: ATCTCCGCGACCTTGTTGAG), *AT4G26410* (fw: GAGCTGAAGTGGCTTCCATGAC, rv: GGTCCGACATACCCATGATCC), *AT4G39670* (fw: GTTCCATACGTGGGCCGTTA, rv: GATAGGTCGCGATGCTTCCA), *AT5G26920* (fw: GTGCCCCAGTGATGAGGTTT, rv: ACCCCTGCACCAATTATGTTG), *AT5G41730* (fw: GAAGTCCAAGCTTAGGTTCAGA, rv: AGGACTCATTGGACTCAAAGGC), and *AT5G42380* (fw: GTAGCGGAAGCAGCTCGTTA, rv: CACCACTTCCTCCACCTCAC).

To visualise the RT-qPCR-derived expression profiles of selected IE genes, the means of the normalized relative expression values from at least two independent experiments were plotted using the pheatmap function (R package pheatmap). For the corresponding heatmaps, genes and samples were clustered according to their averaged relative expression levels, using complete linkage hierarchical clustering with the Euclidean distance as distance measure.

Motif discovery and enrichment analysis

Promoter element enrichment analysis was performed using several tools: MEME-Lab (Brown *et al.*, 2013) was used to find the eight most enriched 6-14 bp elements in promoters of 600 bp length. Scope motif finder was used to find enriched elements in promoters of 1000 bp length, based on BEAM, PRISM, and SPACER programs (http://genie.dartmouth.edu/scope, (Carlson *et*

al., 2007). Weeder1.4 and Weeder2 were run with default settings to identify enriched elements in promoters of 1000 bp length (Pavesi *et al.*, 2006; Zambelli *et al.*, 2014). Pscan was used to assess the enrichment of already known transcription factor binding sites in promoters of 1000 bp length, based on the Jaspar 2016 database (Zambelli *et al.*, 2009; Mathelier *et al.*, 2015). The different outputs were compared to identify motifs consistently and independently picked up by different methods. The RSAT DNA pattern matching tool was used to find occurrences of the selected motifs in the 500-bp regions directly upstream of the transcription start site for the gene set of interest and for the complete set of all *A. thaliana* promoter sequences used as background reference (TAIR10_upstream_1000_20101104.fas or TAIR10_upstream_500_20101028.fas) (Medina-Rivera *et al.*, 2015). The resulting occurrence data was used to calculate the enrichment false discovery rate (FDR) by applying a cumulative hypergeometric distribution with Benjamini-Hochberg correction for multiple testing in R (p.hyper and p.adjust functions). For representation of the motif frequency along the 5' regulatory sequences, the function seqPattern from the seqPattern package in R was used, based on sequences extracted from TAIR10_upstream_1000_20101104.fas and TAIR10_seq_20110103_representative_gene_model_updated.fas.

GO term enrichment analysis

Gene ontology (GO) term enrichment analysis was performed using the agriGO (Du *et al.*, 2010) web tool with default settings for *Arabidopsis thaliana*.

Pathogen infections

Conidiospores of *Blumeria graminis* f. sp. *hordei* (isolate K1 or A6) were inoculated onto the abaxial side of detached leaves placed on agar plates containing 1 mM benzimidazole. The agar plates sealed with surgical tape were placed in a phytochamber (10 h : 14 h, light : dark cycle at 22°C, 60% relative humidity) until sample collection. The Tape-Arabidopsis Sandwich method (Wu *et al.*, 2009) was applied to obtain the abaxial epidermis from the leaf. Approximately 20 leaves per condition were used. The abaxial epidermis with tape was ground in a mortar using a pestle with liquid nitrogen and total RNA was extracted with the Plant RNeasy kit (Qiagen) with the following modifications: the ground powder was mixed with 1.8 ml of the lysis buffer by vortex. Immediately after vortex, the lysate was centrifuged to separate the tape from the lysate. The remaining lysate was used for total RNA extraction according to the manufacturer instructions. Preparation of and inoculation with *Pseudomonas syringae* pv. DC3000 (*Pst*) expressing AvrRpm1, AvrRps4 or neither of these effectors was performed as described before (Liu *et al.*, 2015). Young fully expanded leaves were syringe-infiltrated with a bacterial solution in 10 mM $MgCl_2$ after adjusting the OD_{600} to 0.05, 0.001, and 0.0001 for ion leakage assay, RT-qPCR, and bacterial growth assays, respectively. *Pst* growth assays were performed as described previously (Liu *et al.*, 2015) with following modifications: NYGA medium (5 g /l bactopeptone, 3 g/l yeast extract, 20 ml/l glycerol, with/without 15 g /l agar) with appropriated antibiotics was used for bacterial culture. Further humidity control by covering the plants was omitted. The infiltrated plants were kept in a phytochamber (10 h : 14 h, light : dark cycle at 22°C, 60% relative humidity) until sample collection.

Syringe infiltration of dexamethasone or flg22 into leaves

Unless otherwise mentioned, expression of transgenes under the dexamethasone-inducible promoter was performed by syringe infiltration of 1 µM dexamethasone (DEX, D1756-1G, Sigma-Aldrich, St. Louis, MO, USA). Treatment with flg22 was performed by syringe infiltration of 100 nM and 1 µM of the synthetic peptides for the protein and bacterial growth experiments, respectively. In both cases, each solution was infiltrated into the youngest fully expanded leaves with a 1 ml needle-less plastic syringe.

Temperature shift RNA-seq experiments

Temperature-shift induction of TNL RPS4 immunity in *35S:RPS4-HS* transgenic plants was as described in (Heidrich *et al.*, 2013). Seeds were sown directly onto pots (10 seeds-pot) covered with a lid, placed for three days at 4 and subsequently transferred to 28°C under short day conditions (10 h : 14 h, light : dark cycle, 60% relative humidity). The lid was removed one week after germination. At 3.5 weeks after germination, plants were transferred to 19°C (10 h : 14 h, light : dark cycle, 55% relative humidity) and sampled at the indicated time points after shift. The temperature shift was conducted at different times of the day to allow simultaneous sampling between 16:00 h and 17:00 h for all time points. Leaf material from 4-6 individual plants was pooled for each sample and used subsequently for RNA extraction and RNA-seq. The experiment was repeated independently three times. Data for the 2 h time point was obtained from an experiment distinct from the later time points (Table S1).

References related to Supporting Information

Brown P, Baxter L, Hickman R, Beynon J, Moore JD, Ott S. **2013**. MEME-LaB: motif analysis in clusters. *Bioinformatics (Oxford, England)* **29**: 1696–1697.

Carlson JM, Chakravarty A, DeZiel CE, Gross RH. **2007**. SCOPE: a web server for practical de novo motif discovery. *Nucleic Acids Research* **35**: W259–W264.

Czechowski T, Stitt M, Altmann T, Udvardi MK, Scheible W-R. **2005**. Genome-wide identification and testing of superior reference genes for transcript normalization in Arabidopsis. *Plant Physiology* **139**: 5–17.

Du Z, Zhou X, Ling Y, Zhang Z, Su Z. **2010**. agriGO: a GO analysis toolkit for the agricultural community. *Nucleic Acids Research* **38**: W64-70.

Gentleman RC, Carey VJ, Bates DM, Bolstad B, Dettling M, Dudoit S, Ellis B, Gautier L, Ge Y, Gentry J, *et al.* 2004. Bioconductor: open software development for computational biology and bioinformatics. *Genome Biology* **5**: R80.

Heidrich K, Tsuda K, Blanvillain-Baufumé S, Wirthmueller L, Bautor J, Parker JE. **2013**. Arabidopsis TNL-WRKY domain receptor RRS1 contributes to temperature-conditioned RPS4 auto-immunity. *Frontiers in Plant Science* **4**.403.

Irizarry RA, Hobbs B, Collin F, Beazer ‐ Barclay YD, Antonellis KJ, Scherf U, Speed TP. **2003**. Exploration, normalization, and summaries of high density oligonucleotide array probe level data. *Biostatistics* **4**: 249–264.

Kim D, Pertea G, Trapnell C, Pimentel H, Kelley R, Salzberg SL. **2013**. TopHat2: accurate alignment of transcriptomes in the presence of insertions, deletions and gene fusions. *Genome Biology* **14**: R36.

Liu X, Sun Y, Kørner CJ, Du X, Vollmer ME, Pajerowska-Mukhtar KM. **2015**. Bacterial Leaf Infiltration Assay for Fine Characterization of Plant Defense Responses using the Arabidopsis thaliana-Pseudomonas syringae Pathosystem. *Journal of Visualized Experiments: JoVE*. **104**: e53364-e53364.

Mathelier A, Fornes O, Arenillas DJ, Chen C, Denay G, Lee J, Shi W, Shyr C, Tan G, Worsley-Hunt R, *et al.* **2015**. JASPAR 2016: a major expansion and update of the open-access database of transcription factor binding profiles. *Nucleic Acids Research*: **44**: D110–D115.

Medina-Rivera A, Defrance M, Sand O, Herrmann C, Castro-Mondragon JA, Delerce J, Jaeger S, Blanchet C, Vincens P, Caron C, *et al.* **2015**. RSAT 2015: Regulatory Sequence Analysis Tools. *Nucleic Acids Research*: **43**: W50–W56.

Pavesi G, Mereghetti P, Zambelli F, Stefani M, Mauri G, Pesole G. **2006**. MoD Tools: regulatory motif discovery in nucleotide sequences from co-regulated or homologous genes. *Nucleic Acids Research* **34**: W566–W570.

Quinlan AR, Hall IM. **2010**. BEDTools: a flexible suite of utilities for comparing genomic features. *Bioinformatics (Oxford, England)* **26**: 841–842.

Ritchie ME, Phipson B, Wu D, Hu Y, Law CW, Shi W, Smyth GK. **2015**. limma powers differential expression analyses for RNA-sequencing and microarray studies. *Nucleic Acids Research* **43**: e47.

Smyth GK. **2005**. limma: Linear Models for Microarray Data. In: Gentleman R, Carey VJ, Huber W, Irizarry RA, Dudoit S, eds. Statistics for Biology and Health. Bioinformatics and Computational Biology Solutions Using R and Bioconductor. Springer New York, 397–420.

Sun T, Zhang Y, Li Y, Zhang Q, Ding Y, Zhang Y. **2015**. ChIP-seq reveals broad roles of SARD1 and CBP60g in regulating plant immunity. *Nature Communications* **6**: 10159.

Wang L, Tsuda K, Truman W, Sato M, Nguyen LV, Katagiri F, Glazebrook J. **2011**. CBP60g and SARD1 play partially redundant critical roles in salicylic acid signaling. *The Plant Journal: For Cell and Molecular Biology* **67**: 1029–1041.

Wu F-H, Shen S-C, Lee L-Y, Lee S-H, Chan M-T, Lin C-S. **2009**. Tape-Arabidopsis Sandwich - a simpler Arabidopsis protoplast isolation method. *Plant Methods* **5**: 16.

Zambelli F, Pesole G, Pavesi G. **2009**. Pscan: finding over-represented transcription factor binding site motifs in sequences from co-regulated or co-expressed genes. *Nucleic Acids Research* **37**: W247–W252.

Zambelli F, Pesole G, Pavesi G. **2014**. Using Weeder, Pscan, and PscanChIP for the Discovery of Enriched Transcription Factor Binding Site Motifs in Nucleotide Sequences. *Current Protocols in Bioinformatics / Editoral Board, Andreas D. Baxevanis ... [et Al.]* **47**: 2.11.1-2.11.31.
